# Marked reduction in demographic rates and reduced fitness advantage for early breeding is not linked to reduced thermal matching of breeding time

**DOI:** 10.1002/ece3.3603

**Published:** 2017-11-07

**Authors:** Debora Arlt, Tomas Pärt

**Affiliations:** ^1^ Department of Ecology Swedish University of Agricultural Sciences Uppsala Sweden

**Keywords:** climate change, environmental deterioration, food abundance, mismatch hypothesis, population decline, reproductive success

## Abstract

Warmer springs may cause animals to become mistimed if advances of spring timing, including available resources and of timing of breeding occur at different speed. We used thermal sums (cumulative sum of degree days) during spring to describe the thermal progression (timing) of spring and investigate its relationship to breeding phenology and demography of a long‐distant migrant bird, the northern wheatear (*Oenanthe oenanthe* L.). We first compare 20‐year trends in spring timing, breeding time, selection for breeding time, and annual demographic rates. We then explicitly test whether annual variation in selection for breeding time and demographic rates associates with the degree of phenological matching between breeding time and thermal progression of spring. Both thermal progression of spring and breeding time of wheatears advanced in time during the study period. But despite breeding on average 7 days earlier with respect to date, wheatears bred about 4 days later with respect to thermal spring progression. Over the same time period, selection for breeding time changed from distinct within‐season advantage of breeding early to no or very weak advantage. Furthermore, demographic rates (nest success, fledgling production, recruitment, adult survival) and nestling weight declined markedly by 16%–79%. Those temporal trends suggest that a reduced degree of phenological matching may affect within‐season fitness advantage of early breeding and population demographic rates. In contrast, when we investigate links based on annual variation, we find no significant relationship between either demographic rates or fitness advantage of early breeding with annual variation in the degree of phenological matching. Our results show that corresponding temporal trends in phenological matching, selection for breeding time and demographic rates are inconclusive evidence for demographic effects of changed phenological matching. Instead, we suggest that the trends in selection for breeding time and demographic rates are due to a general deterioration of the breeding environment.

## 1 INTRODUCTION

Timing of reproduction within a season is an important fitness factor for most organisms. The timing of life cycle events (phenology) of many organisms has, however, been altered by global warming (Parmesan, 2007; Walther et al., 2002). Because warmer springs may cause the degree of phenological change to vary among organism groups and trophic levels (Both, Van Asch, Bijlsma, Van Den Burg, & Visser, 2009; Fabina, Abbott, & Gilman, 2010; Visser & Both, 2005), this may cause changes in phenological matching, species interactions, and community composition (Walther, 2010; Walther et al., 2002; Young & Rudolf, 2010).

Given the importance of food resources for reproduction and survival (Martin, 1987; Thomas, Blondel, Perret, Lambrechts, & Speakman, 2001; Visser, Holleman, & Gienapp, 2006), the synchrony between the phenology of breeding and phenology of resources (e.g., food abundance) is expected to have fitness consequences (Durant, Hjermann, Ottersen, & Stenseth, 2007; Miller‐Rushing, Høye, Inouye, & Post, 2010). Many bird species rely on invertebrates (especially arthropods) for feeding young, and the timing of arthropod abundance is closely related to temperature due to the strong temperature dependence in arthropod physiological processes (e.g., growth rates, emergence time; Bale et al., 2002; Trudgill, Honek, Li, & Van Straalen, 2005). Therefore, in seasonal environments warmer springs (i.e., an advanced progression of thermal spring) cause a phenological advancement for arthropods (Bell et al., 2015; Hodgson et al., 2011; Karlsson, 2013; Parmesan, 2007; Robinet & Roques, 2010; Roy & Sparks, 2000). Consequently, if breeding phenology does not keep pace with such changes in the progression of spring, a reduced synchrony may lead to a reduced phenological match between consumers and their food resources (Both et al., 2009; Durant et al., 2007; Sanz, 2003; Visser, van Noordwijk, Tinbergen, & Lessels, 1998). Such a reduced match can change selection for breeding time and have negative consequences for reproduction and survival (Both, Bouwhuis, Lessels, & Visser, 2006; Visser et al., 2006; Durant et al., 2007; Charmantier et al., 2008; Reed, Jenouvrier, & Visser, 2013; but, for other consequences see Miller‐Rushing et al., 2010; Lof, Reed, McNamara, & Visser, 2012; Johansson, Kristensen, Nilsson, & Jonzén, 2015) with consequences for population growth rates (cf. Miller‐Rushing et al., 2010).

Changes in phenological matching between timing of breeding and food resources have been shown to result in changes in selection patterns for breeding time: The advantage of breeding early in the season may be either increasing (Both & Visser, 2001; Gienapp & Bregnballe, 2012; Husby, Visser, & Kruuk, 2011; Reed, Jenouvrier et al., 2013; Visser et al., 1998) or decreasing (Charmantier et al., 2008; Visser et al., 2015), with the direction of change at least partly depending on the prior match and on the direction of the phenology shift (see also Both, 2010). Hence, given the link between the thermal progression of spring and the phenology of arthropods we expect a changed match between the timing of breeding and thermal progression of spring to change patterns of within‐season advantage for early breeding.

The thermal progression of spring can be estimated using thermal sums calculated as the cumulative sum of daily mean temperatures over a time period (see Methods). Such thermal sums have been shown to predict arthropod phenology (Hodgson et al., 2011; Jarošík, Honěk, Magarey, & Skuhrovec, 2011; Lindblad & Sigvald, 1996; Valtonen, Ayres, Roininen, Pöyry, & Leinonen, 2011) and arrival (Saino et al., 2011) and breeding phenology of birds (Charmantier et al., 2008; Kluyver, 1952).

We investigated the links between the thermal progression of spring, breeding phenology, and individual‐ and population‐level demography for an insectivorous, tropical migrant, the northern wheatear (*Oenanthe oenanthe* L.) during 20 years of study. As many other species (Verhulst & Nilsson, 2008), northern wheatears show a general seasonal decline in fitness (i.e., selective advantage for early breeding) due to deteriorating environmental conditions likely involving declines in food availability (Öberg, Pärt, Arlt, Laugen, & Low, 2014; Pärt, Knape, Low, Öberg, & Arlt, 2017). First, we analyzed 20‐year trends of thermal progression of spring and breeding phenology and investigated whether wheatears advanced timing of breeding in relation to an advanced thermal progression of spring. We compared those trends to corresponding trends in within‐year patterns of selection for breeding time and annual demographic rates. Second, to explicitly test whether the degree of phenological matching between breeding time and thermal progression of spring is linked to breeding time selection and demographic rates, we tested whether annual estimates of the degree of matching between timing of breeding and the thermal progression of spring were associated with annual variation in estimates of the advantage of breeding early and demographic rates.

**Figure 1:**
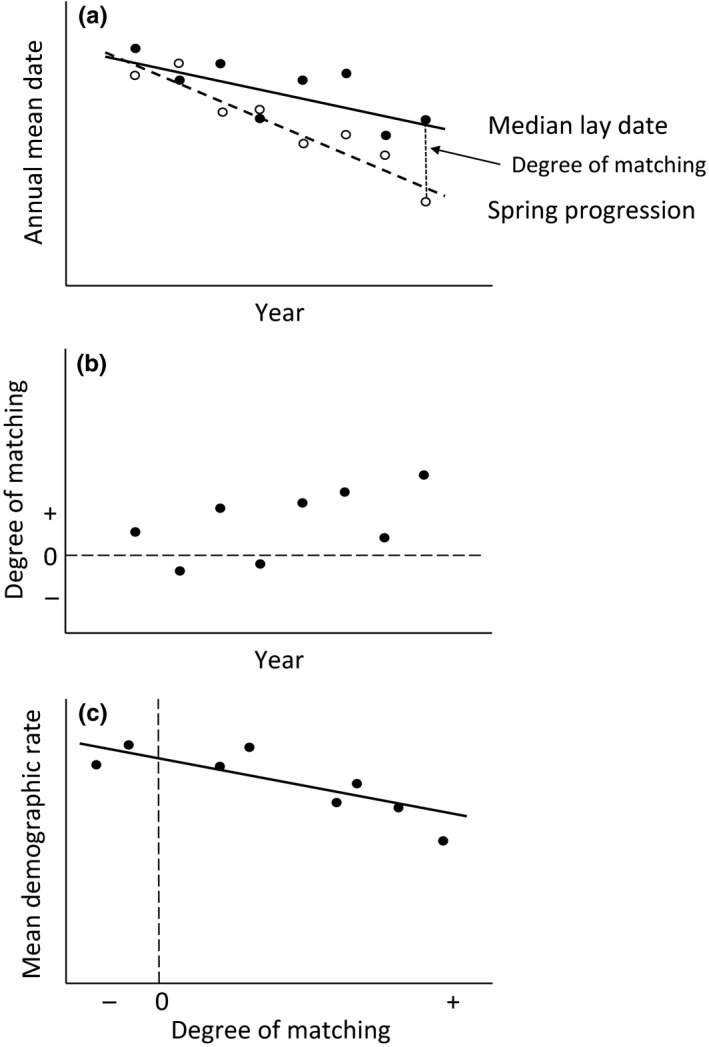
Conceptual illustration of long‐term changes deriving from underlying annual variation in thermal spring progression and breeding phenology, the matching between these two and demographic rates. (a) Spring progression (open symbols) and median lay dates (filled symbols) vary between years but show temporal trends (dashed and solid lines, respectively). The degree of matching in each year is the difference in time between median lay dates and spring progression, exemplified by a dashed vertical line between the data points for the last year. An on average faster advancement of spring progression as compared to the advancement of breeding dates leads to a change in the average degree of phenological matching (difference between the trend estimates increases over the years). (b) The between‐year variation in the degree of phenological matching measured by the difference in days between annual median lay dates and annual estimates of spring progression (as shown in a): median lay dates–spring progression, where larger absolute values correspond to a greater difference in timing (difference can be negative if estimate of breeding time is earlier relative to the reference estimate of spring progression). (c) If there is a direct link between the annual degree of matching and the demographic rates in each year, then we expect a relationship between annual mean demographic rates and the degree of phenological matching, where reduced matching may be expected to result in lower demographic rates

When thermal spring advances faster than the timing of breeding (Figure 1a), we hypothesized that the within‐season advantage of early breeding would change and that delayed breeding with respect to the thermal progression of spring may result in lower annual average rates of reproduction and or survival across the years of study. If the patterns revealed by long‐term trends were supported by the underlying relationships within years, we expected the annual degree of thermal matching (i.e., the birds’ breeding time relative to the thermal progression of spring, schematically illustrated in Figure 1b) to be associated with the strength of within‐season advantage for early breeding (selection for breeding time) and the demographic rates in each year (Figure 1c).

## 2 MATERIALS AND METHODS

### 2.1 Study species and population

Northern wheatears (hereafter wheatears) are small, long‐distant migrant passerines wintering south of the Sahara. They are ground foraging birds with a main distribution in habitats consisting of sparse ground vegetation, being generalists feeding on a range of, mainly, arthropods (primary diet consisting of prey items belonging to *Coleoptera*,* Hymenoptera*,* Lepidoptera*,* Orthoptera*,* Hemiptera*,* Diptera*,* Araneae*; Cramp, 1988). For feeding nestlings, they rely to a large extent on insect larvae (frequently *Lepidoptera*,* Diptera*,* Coleoptera*; Cramp, 1988; van Oosten, 2016; D. Arlt & T. Pärt, unpublished data), and food has been shown to be a limiting factor for wheatear fitness (Seward, Beale, Gilbert, Jones, & Thomas, 2013). We use data from a long‐term population study of wheatears (20 years, 1993–2012) breeding in a heterogeneous agricultural landscape in southern central Sweden (59°50′N, 17°50′E), where they occupy a mosaic of farmland habitats (pastures, farmyards, crop fields, unmanaged grassland; Arlt, Forslund, Jeppsson, & Pärt, 2008). Territories were characterized by vegetation structure describing the height of the ground vegetation layer (field layer height) and categorized as having either a short (<5 cm throughout the breeding season) or tall field layer (growing >15 cm during late incubation and nestling care; Pärt, 2001; Arlt & Pärt, 2007). Wheatears prefer foraging in habitat patches with short field layers (Cramp, 1988; Tye, 1992; own observations), which are positively related to prey availability (Tye, 1992). Reproductive performance and subsequent adult survival is lower for pairs breeding in tall as compared to short field layers (Arlt & Pärt, 2007; Arlt et al., 2008; Low, Arlt, Eggers, & Pärt, 2010; Pärt, 2001). Breeding time was defined by lay date, that is, the date the first egg was laid. For more details of our study area, study population, and basic procedures for data collection, see Supporting Information.

### 2.2 Demographic variables and nestling condition

All demographic data, used to derive the links between breeding time and fitness, were based on data from the central 40 km^2^ part of our study area where on average 90 pairs breed every year (range 55–126). This allowed us to reduce effects of a limited study area on estimates of recruitment and adult survival (Doligez & Pärt, 2008) because we monitored all individuals dispersing within 2–6 km from the central area. Adults disperse short distances between breeding seasons (median distance males: 308 m, females: 352 m; Arlt & Pärt, 2008), and annual resighting probability was high (males: 0.98, females: 0.89; Low et al., 2010). All potential breeding sites of wheatears in the central part of our study area were monitored every third to fifth day throughout the breeding season (see also Supporting Information). We are therefore confident we found all breeding attempts in the central part of the study area, including renesting attempts and second broods.

We used reproductive data from first nest attempts. After nest failure, some pairs lay replacement clutches (average 20%). True second broods after successful nests were rare (0–3 per year). Analyzing total seasonal reproductive success, that is, including renesting attempts and second broods, did not qualitatively change results (details not shown). We analyzed nestling weight (for nestlings aged 5–7 days old) as a proxy of nestling condition and the following demographic variables: nest success (failed vs. successful, i.e., 0 vs. ≥1 fledgling), number of fledglings, number of local recruits, and adult male and female apparent survival (for details see Supporting Information). In our estimates of fledgling and recruit production, we included data from nest attempts that failed after hatching, which is justified because effects of reduced food availability may not only be reflected by a reduced number of offspring but also increased probability of nest failure (e.g., Duncan Rastogi, Zanette, & Clinchy, 2006). Nests that failed before hatching were excluded because the primary reason for those failures was not linked to food availability (predominantly due to predation of eggs and adults; Low et al., 2010; including those failures did not qualitatively change results).

All results were compared to results using a data subset only containing successful nests, that is, restricting analyses to nests for which parental ability to provide nestlings with food is the main critical factor for reproductive and survival parameters. In our study population, about 30% of all nests fail, the majority due to nest predation (about 75% of all failures).

### 2.3 Climatic variables

Data on daily mean temperature and daily precipitation were collected at the Ultuna Climate Station located approximately 10 km from the center of our study area (59°82′N, 17°65′E; http://grodden.evp.slu.se/slu_klimat/index.html. accessed 14.11.2013).

#### 2.3.1 Thermal sum

Plant and arthropod development and hence phenology is strongly determined by degree days (DD), that is, days with influential temperature exceeding a threshold base temperature where development rate is zero, and therefore, insect phenology models are based on thermal sums based on accumulated DD (Hodgson et al., 2011; Jarošík et al., 2011; Nietschke, Magarey, Borchert, Calvin, & Jones, 2007; Nizinski & Saugier, 1988; Valtonen et al., 2011). Here, we used accumulated DD to describe the progression of spring. For each day, we calculated DD as DD = *T*_mean_ − *T*_base_, where *T*_mean_ = daily mean temperature, *T*_base_ = base temperature). We then calculated thermal sums for each day as the accumulated sum of positive degree days starting on January 1. Because the development of different arthropod species is best predicted by different base temperatures, we calculated thermal sums for a range of *T*_base_ between −5°C and +10°C (Hodgson et al., 2011; Valtonen et al., 2011) and investigated which thermal sum (which *T*_base_) best predicted wheatear breeding time.

#### 2.3.2 Precipitation

The amount of rain during the nestling period affects fledging success, recruitment success, and adult survival (Öberg et al., 2015). We used the number of days with rainfall (>0 mm) during the 16 days of the nestling period (from hatch date to fledging) as covariate in analyses of nestling weights and demographic variables.

### 2.4 Derived variables

#### 2.4.1 Phenological matching

The degree of matching between breeding time and the thermal progression of spring (thermal estimate of phenological matching) may be measured in different ways, for example, expressed as the time difference between the matching events or as the timing of a phenological event (e.g., breeding time) measured by the state of the environment at the time of the event. Here, we use both the time difference (in days) between an indicator of the progression of spring and average wheatear breeding time, and timing of breeding measured as thermal sum at breeding. Although a time difference is a more intuitive measure, our measure of thermal sum at breeding is a simpler, more direct measure.

#### 2.4.2 Progression of spring

As an indicator of the progression of spring in each year, we used the date on which a critical thermal sum was reached. Because we were interested in which thermal, sums were most relevant to the timing of breeding of wheatears we investigated which thermal sum best predicted annual median breeding time of wheatears (cf. Ahola, Laaksonen, Eeva, & Lehikoinen, 2012). To find this thermal predictor of breeding time, that is, the thermal sum that best predicted wheatear breeding time, we first derived dates for a range of thermal sums using different *T*_base_. We considered thermal sums reaching values of 100, 200, etc., increasing in steps of 100 until a maximum that corresponded to the thermal sum reached during the end of egg laying period. The thermal sum at the end of egg laying period differed depending on *T*_base_ used in the calculation of DD. We then regressed wheatear annual median lay date against the annual dates at which a certain thermal sum was reached and determined the best thermal predictor of wheatear breeding time based on *R* ^2^ values (i.e., the regression explaining the largest proportion of the variance in breeding time). Across years, wheatear median lay date was best predicted by the date at which a thermal sum of 200 based on DD with *T*_base_ = 3°C (dateTS2003b, hereafter progression of spring) was reached (explaining 78% of the annual variation in lay date; see Results; Fig. S1).

#### 2.4.3 Individual timing: Thermal sum at breeding

An individual's timing relative to temperature (i.e., its thermal matching) may describe its timing relative to the phenology of arthropod food (e.g., Emmenegger, Hahn, & Bauer, 2014; Saino et al., 2011). We calculated the thermal sum (using *T*_base_ = 3°C) for each nest's lay and hatch date (hereafter individual thermal sum), the former likely related to the determination of egg laying (Öberg, 2014), while the latter may more closely relate to the amount of food when resource demand is highest (i.e., during nestling provisioning). This individual measure of thermal matching (individual thermal sum at breeding) can be summarized as an annual population‐level degree of matching (e.g., median thermal sum at breeding), and corresponds in concept to the degree of matching estimated as the time difference between our indicator of the progression of spring and population median lay date (as illustrated in Figure 1b). Both estimates of annual average phenological matching, median thermal sum at lay date and time difference in days between median lay date and dateTS2003b, were strongly correlated (*r* = .944, *t* = 12.08, *df* = 18, *p* < .001).

### 2.5 Within‐season fitness patterns

To assess potential breeding time effects on demography, we investigated slopes (selection for breeding time) and intercepts of the relationship between demographic rates (fitness parameters) and breeding time in each year, using data from first attempts including nests that failed after hatching (see above, Supporting Information for more details). We used a generalized linear model (GLM) with nestling weight or any of the demographic rates as response variable and lay date as explanatory variable. We used models with linear date effects as they fit the data better than models with nonlinear date effects (Supporting Information). We expressed lay dates as relative to the earliest lay date within each year, to make the intercept reflect the performance of the earliest breeder as a reference point. We present slope estimates from models without covariates which are more comparable to estimates reported in previous studies on phenological matching. Because we were specifically interested in extracting effects of thermal spring progression relating to varying resource abundance, we also estimated slopes from models that included covariates known to affect demographic variables and hence within‐season fitness patterns (territory field layer height, female age and amount of rainfall during the nestling period; Arlt & Pärt, 2007; Arlt et al., 2008; Öberg et al., 2014, 2015).

### 2.6 Analyses

Temporal trends of annual average breeding time (median lay date) and within‐season fitness pattern (slope) were analyzed using weighted regression with year as continuous variable and weighted for sample size‐related uncertainty by 1/*SE* (standard error of the annual estimate; sample size varied among years). Trends of thermal sums at the time of breeding (egg lay date or hatching date) in relation to either annual median breeding time or across years were analyzed using data from individual breeding attempts during 20 years and generalized linear mixed models (GLMM), including a random intercept for year to account for the nonindependence of data within years, and individual breeding date as covariate: *y* ~ date + median date + (1|year), or y ~ date + year + (1|year).

Temporal trends of demographic rates and nestling weights were analyzed using individual data and GLMM with random intercepts to account for the nonindependence of data for year, territory site, and female identity (or male identity for male survival analysis). Those models include covariates known to influence demographic rates (i.e., lay date, territory field layer height, female age, and amount of rainfall during the nestling period) and also accounted for potential density effects by including population size (number of established territories in constant study area in each year): *y* ~ year + lay date + female age + rainfall + population size + (1|year) + (1|territory) + (1|individual). The nestling weight model also accounted for nestling age and brood size.

Similar models were used for testing the link between our estimate of annual average phenological matching and annual demographic rates or nestling weights, using annual median of individual thermal sums at time of breeding (population‐level degree of matching) instead of year as a continuous predictor, and individual thermal sums at breeding date (individual‐level matching) instead of lay date. We also tested for quadratic effects but those were not found to improve model fit. Results were qualitatively similar when we used the time difference in days between median wheatear lay date and progression of spring as an alternative estimator for annual average phenological matching (both were strongly correlated, see above; details not shown). For details of the GLMM, see Supporting Information. All analyses were performed using the R software (R Core Team 2013), using the functions “glm” for weighted regressions and “lmer” for GLMMs (package lme4; Bates, Maechler, Bolker, & Walker, 2012).

### 2.7 Ethics statement

Our study was carried out in accordance to the legal and ethical requirements for animal research and welfare. Birds were captured and marked with permission from the Swedish Bird Ringing Centre, Swedish Museum of Natural History (Permit No. 509).

## 3 RESULTS

### 3.1 Trends in spring temperatures and breeding time

**Figure 2:**
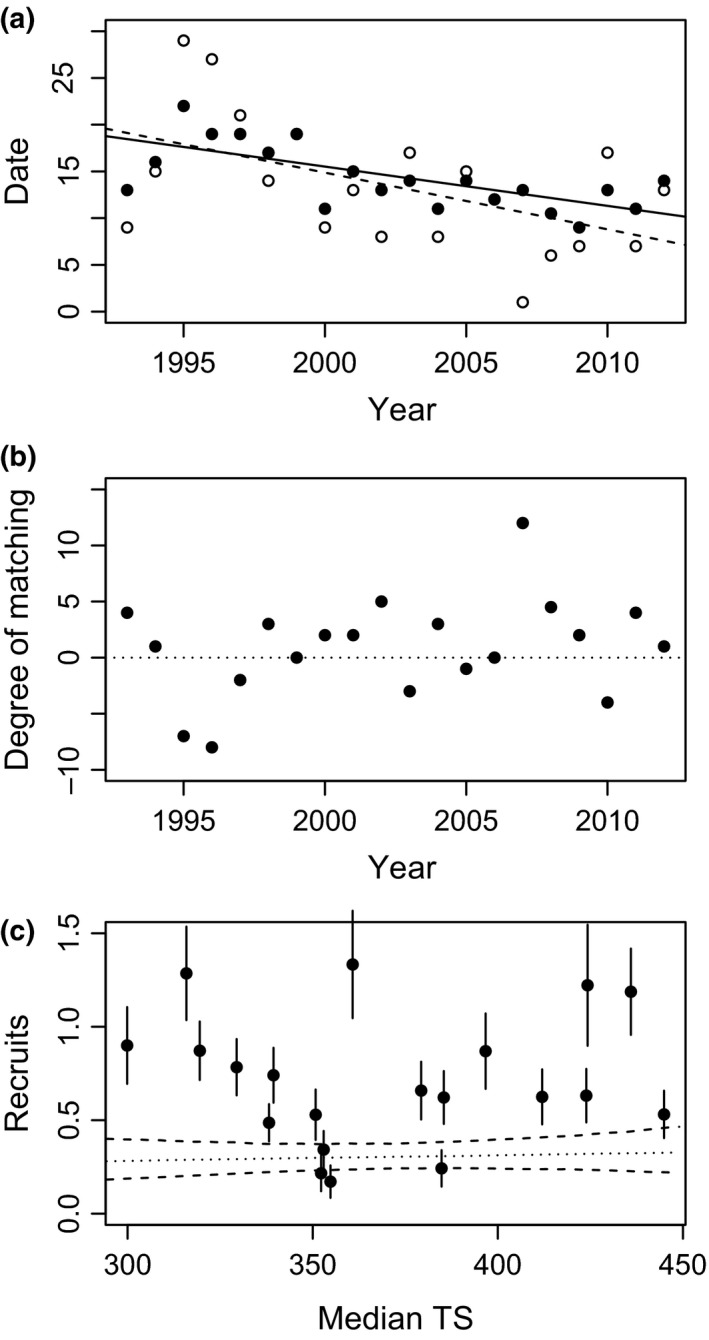
Relationships between the timing of spring temperatures and timing of breeding in wheatears. (a) Temporal trends (years 1993–2012) for thermal progression of spring (open symbols show date TS200b3, that is, the date when thermal sum based on *T*_base_ = 3°C reached 200, dashed line shows linear regression: date TS200b3 ~ year) and median lay date (filled symbols, solid line shows weighted linear regression: median lay date ~ year, weight = 1/*SE*_lay date_). The difference between median lay date and thermal progression of spring describes the degree of phenological matching. (b) Annual estimates of the degree of matching between wheatear timing of breeding and thermal progression of spring resulting from the difference in point estimates for data shown in a. Absolute larger values correspond to a greater difference in timing, where positive values correspond to a relative later timing and negative values to a relative earlier timing of breeding with respect to the estimate for thermal progression of spring. All dates are dates since May 1. (c) There was no direct link between wheatear demographic rates and the annual degree of matching as exemplified for number of local recruits. Analysis details for this and all other demographic rates are shown in Table 4. Median TS, the yearly median thermal sums at hatch date, was estimated from individual thermal sums at hatch date and represents the annual degree in population‐level phenological matching. Dots show mean values, and error bars show *SE* of the raw data. Lines show the predicted relationship generated using bootstrapping implemented in the R package “ez” (see Supporting Information; solid: median, dashed: 95% CI)

There was a close relationship between the timing of spring temperatures in our study area and timing of breeding in wheatears: annual variation in our estimate of the thermal progression of spring (i.e., the date when our critical thermal sum of 200 with *T*_base_ = 3°C was reached, date TS200b3) proved a strong predictor of breeding time explaining 78% of the variation in annual median lay date (linear regression, median lay date~ progression of spring: estimate = 0.424 ± 0.052 *SE*,* t* = 7.998, *p* < .0001, *R* ^2^ = 0.780; Fig. S1). During the 20‐year study period (1993–2012), the timing of spring and breeding have both advanced in time. Our point estimate of the thermal spring timing varied between May 2 and May 30, and advanced by an estimated 11.5 days during the study period (linear regression, progression of spring~year: estimate = −0.607 ± 0.241 *SE*,* t* = −2.520, *p* = .021, *R* ^2^ = 0.261; Figure 2a). Median lay dates of wheatears varied between May 9 and May 22, and advanced by an estimated 7.4 days (linear regression, median lay date~year: estimate = −0.420 ± 0.101 *SE*,* t* = −3.886, *p* = .001, *R* ^2^ = 0.456; Figure 2a). Thus, despite having advanced their median date of breeding by on average a week (7.4 days), northern wheatears bred on average about 4 days later relative to thermal spring progression, which had advanced even more (11.5 days). Around this average change, there was considerable annual variation in the degree of matching between thermal progression of spring and median lay dates (Figure 2b).

This delay relative to thermal spring progression could also been seen when measured for individual nesting attempts: There was a clear trend that wheatears, despite breeding earlier in years with warmer springs, bred delayed relative to thermal spring progression; that is, they bred at higher thermal sums. Individual thermal sums at lay date were about 100–150 DD higher in years wheatears bred early compared to years they bred late (GLMM, trend estimate trend evaluated by LRT test, estimate for median lay date = −13.57 ± 2.06 *SE*,* t* = −6.58, ΔlogLik = 12.2, χ^2^ = 24.5, *df* = 1, *p* < .0001, marginal *R* ^2^ = 0.669), and increased about 80 DD across the study period (estimate for year = 4.47 ± 1.94 *SE*,* t* = 2.30, ΔlogLik = 2.5, χ^2^ = 5.17, *df* = 1, *p* = .024, marginal *R* ^2^ = 0.194; Fig. S2). Results were similar for individual thermal sums at hatch date (Fig. S2).

### 3.2 Trends in within‐season fitness patterns

There is a general pattern of a seasonal decline in reproductive parameters across all years in this population (Öberg et al., 2014), but this advantage of early breeding varied among years (Fig. S3–S8). For all demographic rates except survival of adult females selection for breeding time weakened, that is, the slopes of the relationship between demographic rates and breeding time tended to have changed from distinct negative slopes (i.e., an advantage of early breeding) in the early years to less negative or even positive slopes in more recent years (Table 1, Fig. S9). Results were similar when using data from successful nests only (Table S1). Thus, when investigating slopes from the within‐season fitness patterns without covariates, that is, slopes comparable to estimates reported in previous studies on phenological matching, we find, similar to those studies, that within‐season fitness patterns have changed over time. When accounting for factors that affected demographic parameters also independently of time during the season (covariates female age, territory field layer height, number of rain days during nestling period) slopes seemed more variable among years and a temporal trend was apparent only for number of recruits (recruits: year estimate = 0.005 ± 0.003 *SE*,* t* = 1.94, *p* = .07, *R* ^2^ = 0.173; all other *p* > .27; Fig. S10).

**Table 1:** Estimated temporal trends for within‐season fitness patterns of wheatears (weighted linear regression: slope~year, *w* = 1/*SE*_slope_, *N* = 20 years, *df* = 19). Within‐season slopes of the relationship between demographic rates and breeding time were estimated using data from first nest attempts (including nests failed after hatching), without covariates (data and estimates shown in Fig. S9)

	Estimate ± *SE*	*t*	*p*	*R* ^2^
Nest success	0.006 ± 0.002	2.84	.001	0.309
Nestling weight	0.004 ± 0.002	1.14	.177	0.099
Fledglings	0.002 ± 0.001	1.51	.150	0.112
Recruits	0.004 ± 0.002	1.86	.079	0.162
Male survival	0.006 ± 0.003	1.97	.064	0.178
Female survival	0.001 ± 0.002	−0.21	.840	0.003

The reduced seasonal declines in demographic rates were mainly caused by a reduced performance of the early breeders as there was a clear negative relationship between the annual estimates of intercepts (reflecting success of the earliest breeders, see Methods) and slopes for all demographic rates and nestling weight (correlation, all *r* ≤ −.75, all *p* ≤ .0001, *N* = 20 years; estimates from models without covariates).

### 3.3 Trends in performance and demography

**Figure 3:**
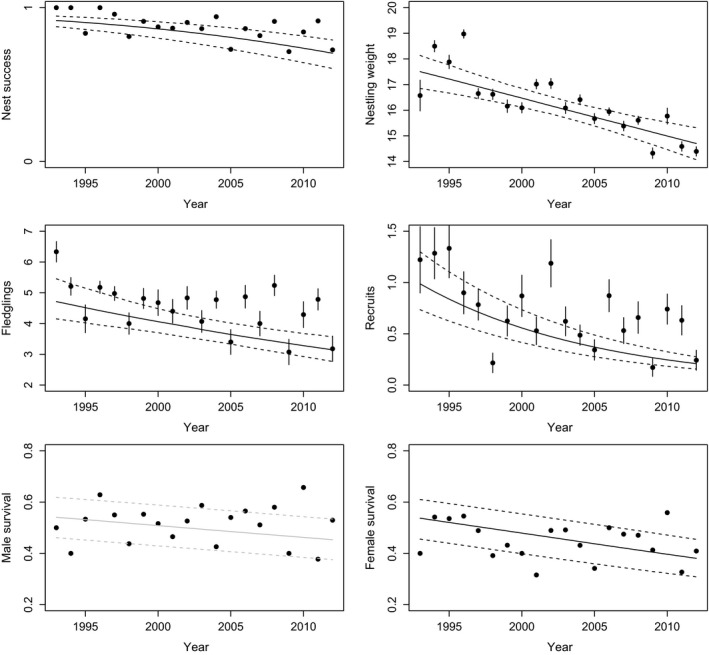
Temporal trends of demographic rates and nestling weight analyzed by mixed models based on results from models in Table 2. Dots show mean values of the raw data, and error bars show *SE* for continuous and count data. Lines show the predicted relationship generated using bootstrapping implemented in the R package “ez” (see Supporting Information; solid: median, dashed: 95% CI). Year trends with *p*‐values ≤.1 are shown by black lines. Due to computational problems using the “ez” package models used to illustrate the predicted relationship do only contain year, but not territory and individual identity, as a random factor

Over the 20‐year study period, we observed strong declines in reproductive and survival parameters across years (Table 2, Figure 3): Nest success has declined on average by a probability of 0.21 (from 0.92 to 0.71), nestling weight by 2.8 g (from 17.5 g to 14.7 g, nestlings aged 5–7 days old), reproduction by 1.56 fledglings (from 4.68 to 3.13) or 0.78 recruits (from 0.99 to 0.21) per nest, and female survival by 0.16 (from 0.54 to 0.38). Relative to the predicted value at the start of the study period the reduction corresponds to 23% for nest success, 16% for nestling weight, 33% for fledglings, 79% for recruits, and 29% for female survival. There was also a tendency for male survival to have declined by 0.09 (from 0.54 to 0.45, i.e., a 16% reduction). Results based on the data only containing successful attempts were qualitatively similar.

**Table 2:** Temporal trends in demographic rates during 20 years (1993–2012) estimated by GLMM using data from first nest attempts (including nests failed after hatching). Temporal trends are shown by the year effects. FLH: territory field layer height (short or tall, estimate for tall), female or male age (young or old, estimate for young), rain: number of days with rainfall >0 mm during the nestling period, density: population size, nest success (successful or failed, estimate for successful). For models analyzing nestling weights *p*‐values were calculated using log‐likelihood ratio tests (all *df* = 1). See Methods for details

	Estimate ± *SE*	*t* or *z*	ΔlogLik^a^	Chi‐square	*p*
Nestling weight (*N* = 2592, *N* nests = 508, *df* = 13, marginal *R* ^2^ = 0.404, conditional *R* ^2^ = 0.787):					
Intercept	6.689 ± 1.227	5.45			
Year	−0.130 ± 0.029	−4.49	8.4	16.8	<.0001
Nestling age	2.213 ± 0.058	38.16	570.2	1140.3	<.0001
Brood size	−0.191 ± 0.054	−3.56	6.1	12.2	.0004
Lay date	0.035 ± 0.014	2.52	426.1	852.6	<.0001
FLH	−0.282 ± 0.148	−1.90	1.6	3.6	.058
Female age	−0.280 ± 0.137	−2.05	1.8	4.1	.043
Rain	−0.082 ± 0.029	−2.80	3.6	7.6	.006
Density	−0.006 ± 0.009	−0.69	0.2	0.5	.486
Nest success (*N* = 874, *df* = 10, marginal *R* ^2^ = 0.103, conditional *R* ^2^ = 0.203):					
Intercept	4.409 ± 1.340	3.29			.001
Year	−0.088 ± 0.032	−2.76			.006
Lay date	0.005 ± 0.019	−0.23			.817
FLH	−0.588 ± 0.244	−2.42			.016
Female age	−0.088 ± 0.231	−0.38			.704
Rain	−0.165 ± 0.054	−3.08			.002
Density	0.002 ± 0.011	0.20			.840
Fledglings (*N* = 716, *df* = 10, marginal *R* ^2^ = 0.102, conditional *R* ^2^ = 0.124):					
Intercept	2.203 ± 0.213	10.35			<.0001
Year	−0.021 ± 0.005	−4.24			<.0001
Lay date	−0.010 ± 0.004	−2.72			.007
FLH	−0.146 ± 0.042	−3.52			.0004
Female age	−0.026 ± 0.041	−0.64			.520
Rain	−0.032 ± 0.010	−3.41			.0007
Density	−0.0004 ± 0.0019	−0.22			.827
Recruits (*N* = 630, *df* = 10, marginal *R* ^2^ = 0.156, conditional *R* ^2^ = 0.262):					
Intercept	2.698 ± 0.651	4.15			<.0001
Year	−0.082 ± 0.016	−5.23			<.0001
Lay date	−0.022 ± 0.011	−2.58			.040
FLH	−0.313 ± 0.121	−2.58			.010
Female age	−0.051 ± 0.118	−0.43			.666
Rain	−0.090 ± 0.027	−3.38			.0007
Density	−0.013 ± 0.006	−2.20			.028
Female survival (*N* = 805, *df* = 11, marginal *R* ^2^ = 0.022, conditional *R* ^2^ = 0.060):					
Intercept	1.095 ± 0.779	1.41			.160
Year	−0.035 ± 0.018	−1.98			.048
Nest success	0.522 ± 0.229	2.28			.023
Lay date	−0.010 ± 0.013	−0.74			.457
FLH	0.036 ± 0.163	0.22			.825
Female age	−0.076 ± 0.175	−0.43			.665
Rain	−0.006 ± 0.034	−0.17			.867
Density	−0.012 ± 0.006	−1.94			.052
Male survival (*N* = 854, *df* = 11, marginal *R* ^2^ = 0.016, conditional *R* ^2^ = 0.035):					
Intercept	1.146 ± 0.756	1.52			.130
Year	−0.020 ± 0.018	−1.18			.240
Nest success	0.361 ± 0.209	1.72			.085
Lay date	0.001 ± 0.013	0.08			.933
FLH	−0.077 ± 0.156	−0.49			.622
Male age	−0.113 ± 0.171	−0.66			.509
Rain	−0.040 ± 0.032	−1.24			.214
Density	−0.009 ± 0.006	−1.47			.143

a Difference: (log‐likelihood of model including predictor of interest) – (log‐likelihood of model without predictor).

### 3.4 Linking annual phenological matching to within‐season fitness patterns and demographic rates

Observed trends across the years of study may suggest effects of changed phenological matching as wheatears were breeding later in relation to the progression of spring, showed reduced seasonal decline in fitness, and showed strong declines in average reproductive and survival parameters. If the patterns suggested by long‐term trends were supported by the underlying relationships within years, we would expect a direct link between the annual degree in phenological matching (i.e., the timing of breeding relative to spring temperature, here estimated as annual median of individual thermal sums at hatching) and within‐season fitness patterns or population demography. However, there was no significant relationship between the annual estimates of either the within‐season advantage for early breeding (slope of the seasonal fitness decline) or demographic rates and the annual degree in phenological matching (slopes: all *p* > .2, Table 3; demographic rates: Table 4, Figure 2c).

**Table 3:** Relationship between within‐season fitness patterns of wheatears (slopes of the within‐season relationship between fitness and breeding date) and the annual median of individual thermal sums (i.e., the timing of breeding relative to spring temperature; median TS) at hatch date (weighted linear regression: slope~ median TS, *w* = 1/*SE*_slope_, *N* = 20 years, *df* = 19). Within‐season slopes of the relationship between demographic rates and breeding time were estimated using data from first nest attempts (including nests failed after hatching), either without covariates, or with covariates age of the breeding female (first year or older), territory field layer height (short or tall), and number of days with rainfall during the nestling period. Results were qualitatively similar, that is, we found no relationships, using the data subset only containing successful nests

	No covariates			With covariates		
	Estimate ± *SE*	*t*	*p*	Estimate ± *SE*	*t*	*p*
Nest success	0.00018 ± 0.00035	0.507	.619	0.00013 ± 0.00099	−0.129	.899
Nestling weight	0.00042 ± 0.00035	1.170	.257	0.0014 ± 0.0007	2.010	.060
Fledglings	−0.00001 ± 0.00014	−0.082	.935	0.00004 ± 0.00022	0.159	.876
Recruits	0.00006 ± 0.00030	−0.212	.840	0.00026 ± 0.00039	0.658	.519
Male survival	0.00015 ± 0.00045	0.334	.740	0.00004 ± 0.00077	0.048	.963
Female survival	−0.00040 ± 0.00030	−1.300	.210	−0.00056 ± 0.00047	−1.193	.248

**Table 4:** Demographic rates in relation to median thermal sums at hatch date (i.e., our thermal estimate for phenological matching) during 20 years (1993–2012), estimated by GLMM using data from first nest attempts (including nests failed after hatching). Median TS, the yearly median thermal sums at hatch date, was estimated from individual thermal sums at hatch date and represents the annual degree in population‐level phenological matching. individual TS: individual thermal sum at hatch date for each breeding attempt (within‐season individual‐level timing with respect progression of spring), FLH: territory field layer height (short or tall, estimate for tall), female or male age (young or old, estimate for young), rain: number of days with rainfall >0 mm during the nestling period, density: population size, nest success (successful or failed, estimate for successful). All models include random intercepts for year, territory site, and female identity (or male identity for male survival analysis). For models analyzing nestling weights, *p*‐values were calculated using log‐likelihood ratio tests (all *df* = 1). See Methods for details. Results based on the data subset only containing successful nests were qualitatively similar

	Estimate ± *SE*	*t* or *z*	ΔlogLik^a^	Chi‐square	*p*
Nestling weight (*N* = 2,592, marginal *R* ^2^ = 0.357, conditional *R* ^2^ = 0.788):					
Intercept	5.292 ± 2.281	2.32			
Median TS	−0.008 ± 0.005	−1.57	1.4	2.68	.102
Nestling age	2.221 ± 0.057	39.10	602.6	1205.1	<.0001
Brood size	−0.196 ± 0.052	−3.77	7.0	14.0	.0002
Individual TS	0.003 ± 0.001	2.02	2.1	4.2	.042
FLH	−0.279 ± 0.143	−1.96	1.9	3.8	.051
Female age	−0.296 ± 0.131	−2.26	11.3	22.6	<.0001
Rain	−0.076 ± 0.032	−2.35	2.9	5.7	.017
Density	0.017 ± 0.013	1.23	0.9	1.7	.191
Nest success (*N* = 874, marginal *R* ^2^ = 0.065, conditional *R* ^2^ = 0.180):					
Intercept	3.043 ± 1.675	1.82			.069
Median TS	−0.0045 ± 0.0042	−1.07			.287
Individual TS	0.0010 ± 0.0019	0.55			.586
FLH	−0.617 ± 0.227	−2.72			.007
Female age	−0.118 ± 0.231	−0.51			.612
Rain	−0.166 ± 0.056	−2.94			.003
Density	0.019 ± 0.010	1.82			.069
Fledglings (*N* = 716, marginal *R* ^2^ = 0.062, conditional *R* ^2^ = 0.118):					
Intercept	1.682 ± 0.358	4.70			<.0001
Median TS	0.0005 ± 0.0008	0.56			.573
Individual TS	−0.0009 ± 0.0004	−2.27			.018
FLH	−0.153 ± 0.042	−3.68			.0002
Female age	−0.031 ± 0.041	−0.76			.447
Rain	−0.023 ± 0.011	−2.19			.029
Density	−0.001 ± 0.002	−0.76			.445
Recruits (*N* = 630, marginal *R* ^2^ = 0.073, conditional *R* ^2^ = 0.270):					
Intercept	0.706 ± 1.361	0.52			.604
Median TS	0.0014 ± 0.0027	0.53			.595
Individual TS	−0.002 ± 0.001	−2.32			.020
FLH	−0.331 ± 0.120	−2.76			.006
Female age	−0.050 ± 0.118	−0.42			.672
Rain	−0.065 ± 0.029	−2.23			.026
Density	−0.003 ± 0.008	−0.45			.650
Female survival (*N* = 805, marginal *R* ^2^ = 0.019, conditional *R* ^2^ = 0.053):					
Intercept	0.804 ± 0.900	0.90			.370
Median TS	−0.0008 ± 0.0023	−0.33			.739
Nest success	0.561 ± 0.227	2.47			.014
Individual TS	−0.0014 ± 0.0013	−1.09			.276
FLH	0.003 ± 0.161	0.02			.987
Female age	−0.054 ± 0.176	−0.31			.760
Rain	0.0004 ± 0.0348	−0.01			.990
Density	−0.008 ± 0.006	−1.35			.176
Male survival (*N* = 854, marginal *R* ^2^ = 0.015, conditional *R* ^2^ = 0.031):					
Intercept	1.080 ± 0.851	1.27			.210
Median TS	−0.0005 ± 0.0021	−0.22			.824
Nest success	0.378 ± 0.208	1.82			.070
Individual TS	−0.0010 ± 0.0013	−0.74			.461
FLH	−0.093 ± 0.154	−0.60			.551
Male age	−0.074 ± 0.171	−0.43			.664
Rain	−0.032 ± 0.033	−0.96			.335
Density	−0.006 ± 0.005	−1.11			.267

a Difference: (log‐likelihood of model including predictor of interest) – (log‐likelihood of model without predictor).

## 4 DISCUSSION

Similar to many other bird species breeding in temperate climate zones (reviewed in Dunn & Møller, 2014), northern wheatears in our study population have advanced their timing of breeding, with as much as about 7 days during the last 20 years. During the same time period, spring phenology advanced even more as the date on which a critical thermal sum was reached (estimate of the thermal progression of spring) has advanced by about 11 days. Thus, despite having advanced their median date of breeding, wheatears still bred on average later in relation to the phenology of spring temperatures. During the same time period, we observed marked negative trends in annual estimates of nestling weight, reproductive output and adult survival (reductions of 16%–79%) and a general tendency of a reduced fitness advantage for early breeding. These trends, in particular the relation between the thermally delayed breeding and reduced performance, are in line with several other studies suggesting a reduced phenological match with respect to timing of spring affecting population performance (Nielsen & Møller, 2006; Saino et al., 2011; Visser et al., 1998). In contrast, capitalizing on information that can be gained from annual variation we found no link between wheatear timing of breeding relative to the thermal progression of spring and either within‐season fitness advantage for early breeding or demographic rates. Our study adds to only few bird population studies directly investigating the link between an estimator of annual average phenological matching (in our case between breeding time and the thermal progression of spring) and annual fitness or demographic rates (Ahola et al., 2012; Charmantier et al., 2008; Dunn, Winkler, Whittingham, Hannon, & Robertson, 2011; Lany et al., 2016; Mallord et al., 2017; Reed, Grøtan, Jenouvrier, Sæther, & Visser, 2013; Reed, Jenouvrier et al., 2013; Vatka, Orell, & Rytkönen, 2011; Visser et al., 2015). Below we compare our results to previous population studies that used detailed demographic data to investigate a possible reduced phenological match and discuss reasons for an absence of population‐level demographic consequences of phenological matching.

### 4.1 Inferring population‐level effects of changes in phenological matching

Suggested negative effects of reduced phenological matching (i.e., decreased synchrony, or increased mismatch) are largely stemming from either single species studies showing a correlation between temporal trends of a measure of phenological matching (or spring temperature) and fitness (measured as demography, selection strength, or population size; e.g., Visser et al., 1998; Both & Visser, 2001; Sanz, 2003; Both et al., 2006; Nielsen & Møller, 2006), or from multispecies studies correlating species differences in the magnitude of phenological shifts with population trends (e.g., Møller, Rubolini, & Lehikoinen, 2008). Such associations of temporal trends can, however, be spurious and caused by other factors than those of interest. Hence, more direct evidence comes from studies linking annual variation in a measure of phenological matching to corresponding annual variation in fitness and demographic rates.

#### 4.1.1 Within‐season fitness patterns

While wheatears are breeding increasingly later with respect to the thermal progression of spring, during the same time period the within‐season advantage of early breeding was reduced to almost being absent during later years. Other studies have shown that when birds bred later in relation to an observed food peak, or earlier in terms of Julian date, phenotypic selection has changed across years from stabilizing or weakly directional to strongly directional selection for early breeding (Both & Visser, 2001; Gienapp & Bregnballe, 2012; Husby et al., 2011; Reed, Jenouvrier et al., 2013; Visser et al., 1998). Yet, other studies have shown either no change (Dunn et al., 2011) or decreased selection (Charmantier et al., 2008). This variation in breeding time selection patterns may be explained by differences in observed synchrony between the phenology of breeding and phenology of resources, hence pattern of selection, at the starting point of a time series (see also Both, 2010). For example, increased directional selection is observed when the starting point was stabilizing selection (Visser et al., 1998, 2006) and decreased selection may be observed when the starting point was a directional selection for early breeding (corresponding to patterns in our study). Differences in trends for selection strength for early breeding may also arise due to other changing factors that affect early and late breeders differentially (see, e.g., Visser et al., 2015).

If changes in selection for breeding time would be mainly driven by changes in phenological matching, then we expect annual variation in selection strength or the relative advantage of early breeding to be related to annual variation in phenological matching. Analyzing the matching to thermal spring progression we found, however, no link between the relative advantage of early breeding in each year and thermal sums at median breeding time. We know of only four studies testing the link between annual average phenological matching and breeding time selection patterns: Charmantier et al. (2008) showed that selection strength for earlier breeding was associated with the degree of synchrony with peak food abundance, while Dunn et al. (2011), Ahola et al. (2012), and Visser et al. (2015) found no such association.

#### 4.1.2 Annual demographic rates

Several studies have shown temporal trends of declining demographic rates (or declining population size), during a time period of increasing spring temperatures, suggesting negative demographic effects of an reduced phenological match (e.g., Both et al., 2006; Gienapp & Bregnballe, 2012; Sanz, 2003). We found strong declines in nestling weight and several demographic rates during our study period, in line with the view of deteriorating conditions for breeding, especially early in the breeding season as the decline was mainly due to reduced performance of the early breeding birds. However, we found no link between thermal sums at median breeding time and annual estimates of our performance and demographic rates. Only five other studies known to us have directly linked annual average phenological matching to annual demographic rates: One suggests that demographic rates have increased with an increased phenological synchrony (Vatka et al., 2011), one found a quadratic relationship between average annual reproductive success and phenological synchrony (Lany et al., 2016), while the other three suggest weak or no links between an variations in phenological matching and demographic rates (Ahola et al., 2012; Mallord et al., 2017; Reed, Jenouvrier et al., 2013; Reed, Grøtan et al., 2013; Visser et al., 2015).

To sum up, although correlated temporal trends of changes in phenological matching and changes in within‐season fitness patterns, demographic rates, or population size may suggest demographic effects of a reduced phenological match, the supportive evidence from links between annual estimates of the degree of phenological matching and demography is still largely missing.

### 4.2 Why a missing link?

Capitalizing on annual variation, we found no relationship between the degree of phenological matching and population demographic rates. The lack of evidence for such a link may have several explanations (see also Reed, Jenouvrier et al., 2013). While demographic compensation (Reed, Jenouvrier et al., 2013) cannot explain our results because we observed similar, or at least not contrasting, results for different demographic rates, the most likely explanations relate to the role of other factors relative to matching resource availability determined by spring timing.

First, environmental stochastic variation in demographic rates (due to variation in environmental conditions, e.g., weather, when feeding young) that is independent of phenological matching may mask demographic effects of phenological matching. The advantage of breeding early may change depending on environmental conditions (e.g., Tarwater & Beissinger, 2013); for example, a seasonal decline in fitness may change to no or a positive relationship because of adverse weather early in the breeding season. Rainfall during the nestling stage affects reproductive output and probability of recruitment in wheatears (Öberg et al., 2015), although rain showed no directional seasonal distribution and including rain data in our models did not change our findings. Demographic variation may also be due to variation in overall level of resource abundance (e.g., food, Durant et al., 2005; which we discuss below), or population density (see e.g., Reed, Jenouvrier et al., 2013; Reed, Grøtan et al., 2013; Tarwater & Beissinger, 2013) but including population density in the statistical models did not change our findings. However, even with long‐term data (most studies are based on sample of 10–30 years), the power to detect a link between an annual average phenological matching and demographic rates may often be low, depending on the magnitude of environmental stochasticity and the ability to account for some of this variation. Hence, an absence of such a link should be taken with care and is no evidence for an absence of an effect of changed phenological matching per se.

Second, changes in relative advantage of early breeding and temporal trends in demography could be caused by a general deterioration of the environment, for example, a change to an overall lower food abundance, or availability, without a distinct peak. Several insect groups show European‐wide population declines that seem related to both climate and land use changes (Dirzo et al., 2014; Potts et al., 2010; Thomas et al., 2004; Hallmann, Sorg, Jongejans, Siepel, Hofland, Schwan, et al., 2017). Furthermore, species‐specific responses to increasing spring temperature (Diez et al., 2012; Karlsson, 2013; Pau et al., 2011) may also change community composition (in terms of relative species abundances, and hence species interactions) at any given time during the season (Parmesan, 2006; Walther, 2010; Diez et al., 2012) and likely affect seasonal availability and thus potentially reduce the height of the peak in arthropod food abundance at the time of nestling feeding. Such temperature‐related changes in average food abundance have been shown to affect demography (Pearce‐Higgins, Dennis, Whittingham, & Yalden, 2010; van de Pol et al., 2010; see also Gienapp & Bregnballe, 2012) and our observed reduced nestling weight, reduced number of fledglings and recruits across the years of our study of wheatears are in line with such a general decline in food abundance or availability. Although we have no data on arthropod food abundance, two other facts suggest general food availability may have declined. First, the general level of nest predation has increased across the study period and increased nest predation risk may reduce the amount of food provisioned to nestlings (e.g., Sofaer, Sillett, Peluc, Morrison, & Ghalambor, 2013; Dudeck, Clinchy, Allen, & Zanette, in press). Second, wheatears may suffer from a reduced availability of food due to increased ground vegetation height when feeding nestlings. Field layer height within the territory is an important determinant of food availability, reproductive output, and adult survival in our population of wheatears (see Methods). Although we partly accounted for ground vegetation height by including territory level field layer class in the statistical models, crude within‐territory estimates of the proportion of short vegetation at the time when most pairs feed nestlings suggest that there has been a reduction in the proportion of short field layer over the 20 years of our study (including grassland and crop habitats; D. Arlt & T. Pärt, unpublished data). Probable reasons for such a change in ground vegetation height are reduced grazing intensity, increased amount of autumn‐sown crops, and advanced timing of vegetation growth. Our results are similar to those of Ahola et al. (2012) and Mallord et al. (2017) who conclude that changes in demographic rates or population size were more likely explained by other factors than timing with respect to resource availability.

## 5 CONCLUSION

Our results show that, despite superficial evidence from correlated temporal trends, direct evidence for population‐level effects of changes in phenological matching between timing of breeding and thermal progression of spring was lacking. A reduced phenological matching with negative demographic consequences as suggested from an association of thermally delayed timing of breeding and temporal declines in demographic rates was not supported by annual variation in those measures: There was no link between the annual degree of phenological matching and either selection for breeding time or annual demographic rates. Correlated long‐term trends of breeding time and selection for breeding time or demography are at best indicative but not conclusive of changes in phenological matching or effects of such changes.

Our results also suggest that other factors may mask relationships between phenological matching and population‐level demography. One interpretation of our results suggests a general deterioration of the environment in terms of food abundance or availability that may cause the observed reduction in demographic rates and reduced advantage of early breeding during the last 20 years. Therefore, predicting demographic impacts of climate change should not only consider phenological matching with a resource, but also other factors, in particular possible changes in habitat quality and general levels of resource abundance (see also Visser et al., 2015).

## CONFLICT OF INTEREST

None declared.

## AUTHOR CONTRIBUTIONS

D. A. and T. P. had equal parts on conceptualizing and designing the work, D. A. analyzed the data and drafted the work, D. A. and T. P. equally contributed to data interpretation and revising the work.

## DATA ACCESSIBILITY

Data supporting the results in this article are available from the Dryad Digital Repository: https://doi.org/10.5061/dryad.qp811 (Arlt & Pärt, 2017).
