# Supplementary material for: Marked reduction in demographic rates and reduced fitness advantage for early breeding is not linked to reduced thermal matching of breeding time
Source: Ecol Evol. 2017 Nov 7;7(24):10782–96. doi: 10.1002/ece3.3603 (PMC5743537; doi:10.1002/ece3.3603)
Supplement: Supplementary file 1 [file ECE3-7-10782-s001.pdf]

Supporting Information

Arlt, D. and Pärt, T.

Marked reduction in demographic rates and reduced fitness advantage for early breeding is not linked to reduced thermal matching of breeding time

1. Extended Methods

1.1 Details on study area and study population

1.2 Nestling condition and demographic variables

1.3 Within-season fitness patterns

1.4 Statistical analyses

2. Relationship between wheatear breeding time and thermal progression of spring

3. Thermal matching based on data from individual nests

4. Within-season fitness pattern

4.1. Plots of demographic variables against breeding time for each year

4.2 Extended results for temporal trends of within-season fitness pattern

1. Extended Methods

1.1 Study area and study population

We use data from a long-term population study of wheatears (20 years, 1993-2012) breeding in a heterogeneous agricultural landscape in southern central Sweden (59°50'N, 17°50'E) in an area of about 60 km^2. At our study area wheatears arrive and establish territories from mid-April to mid-May, the first pairs start egg laying in early May, and the majority of nestlings fledge before mid-June (details in Pärt 2001, Arlt & Pärt 2007). Each year we monitored all sites potentially suitable for wheatears from early April to the end of June. Detailed breeding and demographic data were collected in a 40 km^2 central part of the study area. In the outer part of the study area we monitored at least site occupancy, nest success and identity of the breeding birds. Nearly all males (97%) and 76% of all females could be aged as either young (one year old) or older (≥ two years old) based on plumage characteristics (see Pärt 2001, Jenni & Winkler 1994). In our study area nest sites are abundant and nests are placed either at the ground under stones (in stone piles and stone walls) or under roof tiles of buildings (ca. 20%, mainly barns).

In the central part of our study area monitoring was normally done every third to fifth day from early April and throughout the breeding season. Because wheatears are often easily observed perching on prominent structures like rocks, fence posts or roofs we are confident to have identified all breeding attempts in this central part of the area. The birds' behaviour allows to identify their status during the breeding season, e.g. whether they initiated a nest, started to incubate, when they started feeding young, and also when a nest got depredated, even when the actual nest is not found yet. Breeding time was characterised by lay dates which were defined as the date the first egg was laid. Lay dates were normally calculated based on the observed age of chicks in the accessible nests (about 90% of all dates, accuracy of 0-1 days). For inaccessible nests lay dates were based on observations of breeding behaviour that could be used to establish likely time intervals for lay dates (e.g. nest building, first observation of feeding parents, age of fledged young; 85% of all interval lengths ≤5 days) and calculated as mean date in the interval. For the calculations of lay date we assumed an incubation period of 13 days (Cramp 1988; T. Pärt & D. Arlt, unpublished data), and start of incubation on the day the penultimate egg was laid. In cases with unknown clutch size (i.e. the nest was found >2 days after hatching) we used a clutch size of six (population mean 6.05±0.77 SD for all years, N=455). Across the 20 year study period median lay date for first breeding attempts was 15 May (10% - 90% percentile: 7 May - 24 May). Hatch date was calculated from lay dates using the above assumptions.

1.2 Nestling condition and demographic variables

Nestling condition: As a proxy of nestling condition we analysed nestling weight as it commonly shows a clear link to food abundance (Brickle et al. 2000, Hart et al. 2006, Siikamäki et al. 1998, Visser et al. 2006). We weighed nestlings to the nearest 0.1 g on the day of ringing, i.e. when 5-7 days old. At this age ageing (based on development of feather tracts) is accurate (based on a subsample of nestlings with known hatch date; D. Arlt & T. Pärt, unpublished data) and we used only weight of nestlings aged 5-7 days. Nestling age and brood size (number of chicks in nest on the day of ringing) was included as covariate in all analyses. Nestling weight is commonly linked to probability of recruitment (Tinbergen & Boerlijst 1990, Lindén et al. 1992, Both et al. 1999). In our study population probability of recruitment increased with nestling weight (GLMM with random slope for year and identity of breeding attempt, and covariates lay date, nestling age,

territory field layer height (see Methods): weight estimate=0.09±0.03 SE, z=3.1, p=0.002, N=3794, 772 nests).

Nest success: Nest success was recorded as successful or failed. A breeding attempt was defined successful when we observed fledglings or heard intense warning calls of the parents after expected date of fledging (Pärt 2001). Nest failures, on average 30%, were mostly due to predation (Pärt 2001). Nest failures were more common in territories with tall field layer (Pärt 2001, Arlt & Pärt 2007, Low et al. 2010), when located near habitat edges with contrasting heights of ground vegetation (Schneider et al. 2012), and later in the season (Öberg et al. 2014). Data on nest success were missing when the nest had not been visited at or after the time of fledging (about 12% of all breeding attempts).

Fledglings: The number of fledged offspring was defined as the number of chicks ringed minus number of dead chicks found in the nest after fledging. Partial nest predation is extremely rare (<1% of all successful attempts with observations of fledglings). Data on number of fledglings was missing due to inaccessibility or missing data on the presence of dead chicks in the nest after fledging (28% of all successful nests).

Recruits: Each year we uniquely colour-ringed nestlings from accessible nest sites (69% of all nest sites), and recruitment was estimated by return of nestlings ringed in the 40 km^2 central area to the entire 60 km^2 area in subsequent years (up to 2014, 95% of local recruits recruited to the population within two years after birth). In our population wheatears display a high degree of philopatry with on average 11% of all marked and fledged juveniles returning to breed in the study area.

Adult survival: Each year we uniquely colour-ringed many adults, resulting in 70-75% of breeding males and females being marked at the end of the breeding season. Apparent adult survival was estimated by return of birds breeding in the central area to the entire area in subsequent years (up to 2014). Although survival may be related to factors outside the breeding season previous results show that adult survival is also influenced by parental workload (Low et al. 2010) and thus may be related to food availability during the chick rearing period (see also Seward et al. 2013).

1.3 Within-season fitness patterns

Wheatears show seasonal declines in fitness with earliest breeders having highest fitness, and we have evidence that seasonal fitness declines in reproductive rates are linked to declines in food abundance or availability (Öberg et al. 2014; T. Pärt et al., unpublished manuscript). To assess the between-year variation of breeding time effects on demography we investigated annual slopes and intercepts of the relationship between demographic rates and breeding time, using data from first breeding attempts.

There were a few pairs that were first observed late in season and that initiated late nests. Because those nests may have been replacement nests by birds that had failed and then moved into our surveyed area, we excluded the few nest for which we were doubtful whether they were true first nests (lay date >24 May, see above) from analyses. About 20% of failed first attempts (nest failure rate is about 30%, see above) are followed by a renesting attempt. Hence, renesting attempts constitute about 6% of all first attempts, and this frequency has not changed over the years. In our population true second attempts after a successful first nest were rare (0–3 per year). After a first nest the birds' behaviour indicated whether they initiated a replacement nest (male seen guarding female, female rarely seen due to incubation) or a true second brood (male is provisioning the fledglings and female rarely seen due to incubation, female does not show

signs of moult 3-4 weeks after fledging of the first nest). Analysing total seasonal reproductive success of females, i.e. including renesting attempts and 2nd broods in cases when outcome for all nesting attempts within a season were known, did not change results qualitatively (details not shown).

We used generalized linear models (GLM) with lay date as continuous variable and nestling weight, nest success, the number of fledglings, the number of recruits, or adult survival as response variable. To extract meaningful intercepts we expressed lay dates as relative to the earliest lay date in each year, i.e. the intercept can be seen as reference point describing the performance of the earliest breeder. For number of fledglings and recruits we used a Poisson distribution with log link function, for nest success and adult survival we used a binomial distribution with logit link function. For each year we saved intercept, slope estimate and the SE for the slope estimate for further analyses.

Our aim was to assess the match between wheatear breeding time and resource abundance. Previous studies have shown that reduced food abundance can increase probability of nest failure during nestling provisioning, e.g. through starvation, or through cues provided to predators by offspring in poor condition or parental activity (Leech & Leonard1997, Martin et al. 2000, Duncan Rastogi et al. 2006, Sofaer et al. 2013). Hence, we included data from nest attempts that failed after hatching in our analyses, maximising the amount of variation in demographic variables potentially related to resource abundance. Nests that failed before hatching (and a few nests that failed due to disturbance by human activity) were excluded. However, if resource abundance affects reproductive and survival parameters mainly via parental ability to provide nestlings with food and less through nest predation we need to focus on successful nests. Therefore we also compared results with results from analyses including successful nests only (excluding all failed nests; majority of nest failures in wheatears were due to nest predation, Pärt 2001).

Furthermore, other factors like parental age, territory quality or environmental condition affect demographic variables. Such factors can potentially be accounted for by including them as covariates in the models; although accounting for covariates does not guarantee that the resulting within-season relationships between fitness and breeding time more directly reflect resource abundance. We therefore also estimated slopes from models including covariates known to affect demographic variables, i.e. territory field layer height, female age and amount of rainfall during the nestling period (Arlt & Pärt 2007, Arlt et al. 2008, Öberg et al. 2014, Öberg et al. 2015).

We tested whether within-season relationships between fitness and breeding time were linear or non-linear (quadratic) by comparing models with a linear date term and models with a quadratic date term based on AIC (Akaike information criterion, Burnham and Anderson 2002). In all cases a linear relationship fit the data better than a non-linear (all $\Delta AIC>2$, details not shown).

1.4 Statistical analyses

Temporal trends of demographic rates and nestling weights - were analysed using individual data and generalised linear mixed models (GLMM) with random intercepts for year, territory site and female identity (or male identity for male survival analysis). Models included year as continuous predictor and covariates that were known to influence demography (Pärt 2001; Arlt & Pärt 2007, Low et al. 2010, Öberg et al. 2014, Öberg et al. 2015). For analyses of demographic rates covariates included were lay date, territory field layer height (short or tall), female age (young or older), amount of rainfall during the nestling period and for analyses of adult survival

also nest success (failed or successful). Models also accounted for potential density effects by including population size (number of established territories in each year). For analyses of reproductive success we used data for male age where female age was missing (about 30% of all ages; male and female age were strongly associated: chi-square=183.1, df=1, $p<0.0001$, N=1321). For analyses of nestling weights covariates included were age of chicks, brood size (number of ringed chicks), lay date, female age, territory field layer height and population size. We tested all two-way interactions between year and covariates, as well as between population size or rainfall and all other covariates. All interaction terms had p-values >0.05 (details not shown). We also tested quadratic effects but all quadratic terms had had p-values >0.05 (details not shown). We used the function ezPredict() in R package 'ez' (Lawrence 2013) to compute the predicted values from the fixed effects of the model. We calculated the change in demographic rates and nestling weights across the 20 years between predicted values (median of the predicted values from 1000 iterations) for the first and last year. We describe the decrease in demographic rates or nestling weights as the change relative to the first year value.

Linking annual phenological matching to within-season fitness patterns and demographic rates - To test whether there was a direct link between annual variation in phenological matching and demographic rates, we investigated whether annual variation in (i) the slope of the within-season relationship between fitness and breeding time, and (ii) demographic rates and nestling weights, was related to annual variation in our estimate of phenological matching, i.e. average individual thermal sums at hatching (estimated as median of thermal sums at hatch date for each breeding attempt; see Methods in main text for details calculating thermal sums). We used similar models as for analyses of temporal trends of demographic rates, i.e. individual data and mixed models with random intercepts for year, territory site and female (or male) identity. Instead of year we used the median of individual thermal sums at hatching as continuous predictor (population-level match), and instead of individual lay date we used individual thermal sums at hatch date (within-season individual-level match). Other covariates were the same. We tested two-way interactions (similar as for analyses of temporal trends) and quadratic effect, but all had p-values >0.05 (details not shown).

For GLMMs with normally distributed response variables (individual thermal sum at the time of breeding, nestling weight) significance tests for individual predictor variables were done using likelihood ratio tests (LRT) based on a comparison of the full model with a reduced model that had dropped the predictor variable in question. The fit of GLMMs was assessed by R^2, with marginal R^2 relating to variance explained by fixed factors, conditional R^2 to variance explained by the full model (Nakagawa & Schielzeth 2013).

References

Arlt D et al. 2008. Habitat-specific population growth of a farmland bird. PLoS ONE 3:e3006.

Arlt D & Pärt T 2007. Nonideal breeding habitat selection: A mismatch between preference and breeding success. Ecology 88: 792-801.

Both C et al. 1999. Density-dependent recruitment rates in great tits: the importance of being heavier. P Roy Soc Lond B 266: 465-469.

Brickle NW et al. 2000. Effects of agricultural intensification on the breeding success of corn buntings *Miliaria calandra*. J Appl Ecol 37: 742-755.

Burnham KP & Anderson DR 2002. Model Selection and Multi-model Inference – a Practical Information Theoretical Approach. 2nd ed. Springer Verlag, New York.

Cramp S 1988. Handbook of the Birds of Europe, the Middle East and North Africa: the Birds of the Western Palearctic. Volume V: Tyrant Flycatchers to Thrushes. Oxford University Press, New York.

Duncan Rastogi A et al. 2006. Food availability affects diurnal nest predation and adult antipredator behaviour in song sparrows, *Melospiza melodia*. Anim Behav 72: 933-940.

Hart JD et al. 2006. The relationship between yellowhammer breeding performance, arthropod abundance and insecticide applications on arable farmland. J Appl Ecol 43: 81-91.

Jenni L & Winkler R 1994. Moult and Ageing of European Passerines. Academic Press, London.

Lawrence MA 2013. ez: Easy analysis and visualization of factorial experiments. R package version 4.2-2. http://CRAN.R-project.org/package=ez

Leech SM & Leonard ML1997. Begging and the risk of predation in nestling birds. Behav Ecol 8: 644-646.

Lindén M et al. 1992. Selection on Fledging Mass in the Collared Flycatcher and the Great Tit. Ecology 73: 336-343.

Low M et al. 2010. Habitat-specific differences in adult survival rates and its links to parental workload and on-nest predation. J Anim Ecol 79: 214–224.

Martin TE et al. 2000. Nest predation increases with parental activity: separating nest site and parental activity effects. P Roy Soc Lond B 267: 2287–2293.

Nakagawa S & Schielzeth H 2013. A general and simple method for obtaining R2 from generalized linear mixed-effects models. Meth Ecol Evol 4: 133–142.

Öberg M et al. 2014. Decomposing the seasonal fitness decline. Oecologia 174: 139-150.

Öberg M et al. 2015. Rainfall during parental care reduces reproductive and survival components of fitness in a passerine bird. Ecol Evol 5: 345–356.

Pärt T 2001. The effects of territory quality on age-dependent reproductive performance in the northern wheatear, *Oenanthe oenanthe*. Anim Behav 62: 379–388.

Schneider NA et al. 2012. Contrast in Edge Vegetation Structure Modifies the Predation Risk of Natural Ground Nests in an Agricultural Landscape. PLoS ONE 7(2): e31517.

Seward AM et al. 2013. The impact of increased food availability on survival of a long-distance migratory bird. Ecology 94: 221–230.

Siikamäki P 1998. Limitation of reproductive success by food availability and breeding time in pied flycatchers. Ecology 79: 1789-1796.

Sofaer HR et al. 2013. Differential effects of food availability and nest predation risk on avian reproductive strategies. Behav Ecol 24: 698-707.

Tinbergen JM & Boerlijst MC 1990. Nestling weight and survival in individual great tits *Parus major*. J Anim Ecol 59: 1113-1127.

Visser M et al. 2006. Shifts in caterpillar biomass phenology due to climate change and its impact on the breeding biology of an insectivorous bird. Oecologia 147: 164-172.

2. Relationship between wheatear breeding time and thermal progression of spring

There was a close relationship: annual variation in our estimate of the thermal progression of spring (i.e. the date when our critical thermal sum of 200 with base temperature T_{base}=3 °C was reached, date TS200b3) was a strong predictor of breeding time explaining 78% of the variation in annual median lay date (weighted linear regression, median lay date~ progression of spring, weight=1/$SE_{lay\ date}$: estimate=0.424±0.052 SE, $t=7.998$, $p<0.0001$, $R^2=0.780$; Fig. S1).

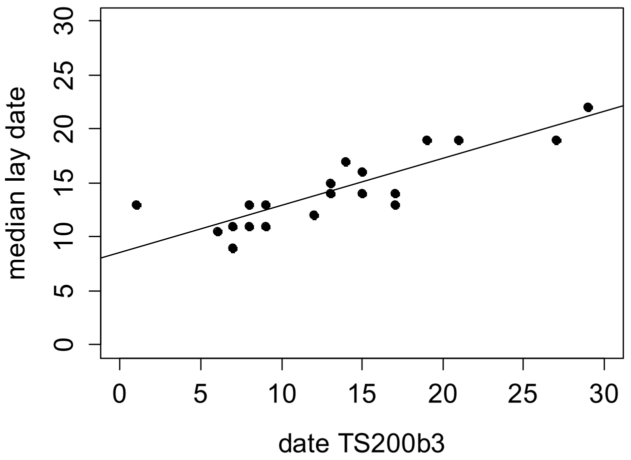

Fig. S1. Relationship between the timing of breeding in wheatears (annual median lay date) and timing of spring temperatures, i.e. the thermal progression of spring (date TS200b3). Thermal progression of spring was estimated as the date when thermal sum based on T_{base}=3°C reached 200. Line shows the estimated relationship from a linear regression.

3. Thermal matching based on data from individual nests

When measured for individual nesting attempts there was a clear trend that thermal matching, i.e. the birds' timing of breeding relative to the thermal progression of spring, had changed: wheatears bred delayed relative to thermal progression of spring (Fig. S2).

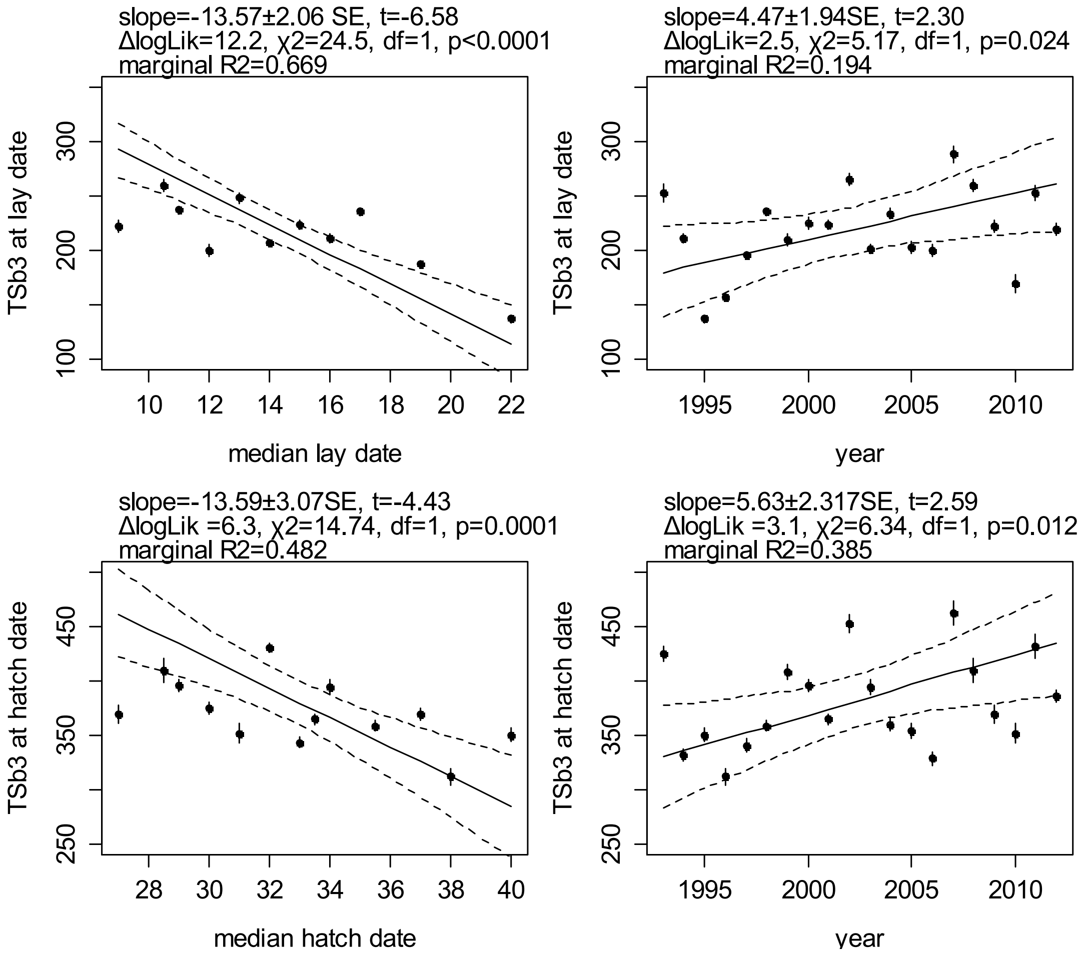

Fig. S2. Trends of individual thermal sums (with T_{base}=3ºC, TSb3) at lay date (top panels) or at hatch date (bottom panels) in relation to annual median lay or median hatch date (relative to 1 May, date 1 = 1 May), and across years. Trends were analysed using data of individual breeding attempts during 20 years and generalised linear mixed models (GLMM), including a random intercept for year, and individual breeding date as covariate: individual thermal sum~individual date+median date+(1|year), or individual thermal sum~ individual date+year+(1|year). Lines show the predicted relationships generated using bootstrapping implemented in the R package 'ez' (see Extended Methods above; solid: median, dashed: 95% CI). Slope of trend evaluated by LRT test. See Methods. For all models N=1319. Points (some overlapping due to shared median lay or hatch dates) show means and SE for raw data.

4. Within-season fitness pattern

4.1. Plots of demographic variables against breeding time for each year

Figures S3-S8 show variation between years in the relationship between different demographic variables and breeding time (lay date).

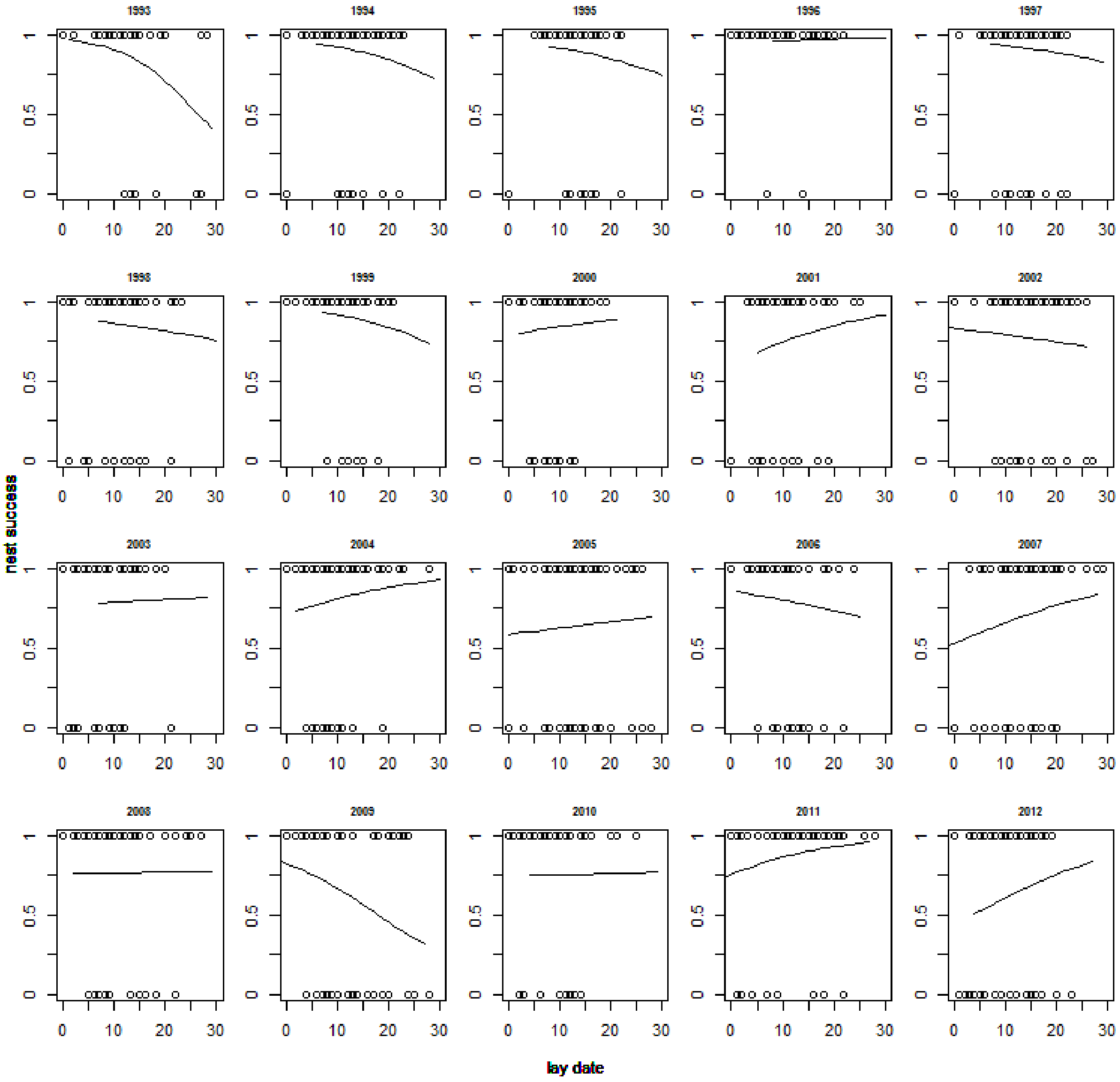

Fig. S3.Seasonal patterns of nest success (0: failed, 1: successful) across 20 years. Lines show predicted probability of nest success from a GLM with binomial distribution (logit link) without covariates. Lay dates are shown as relative to the earliest lay date in each year.

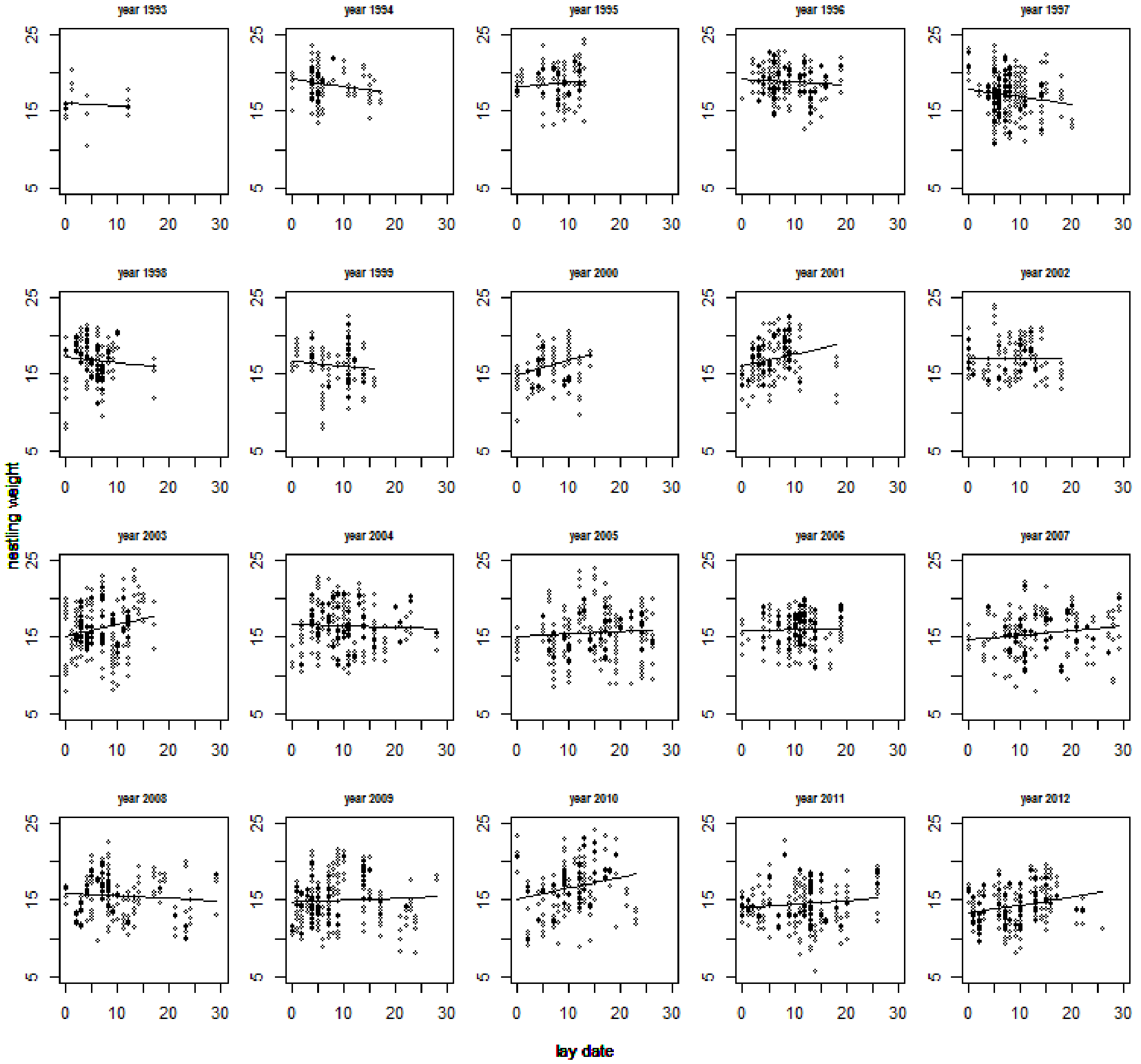

Fig. S4. Seasonal patterns of nestling weights (in gram, nestlings age 5-7 days old) across 20 years. Lines show predicted nestling weight from a linear model with normal distribution without covariates. Lay dates are shown as relative to the earliest lay date in each year.

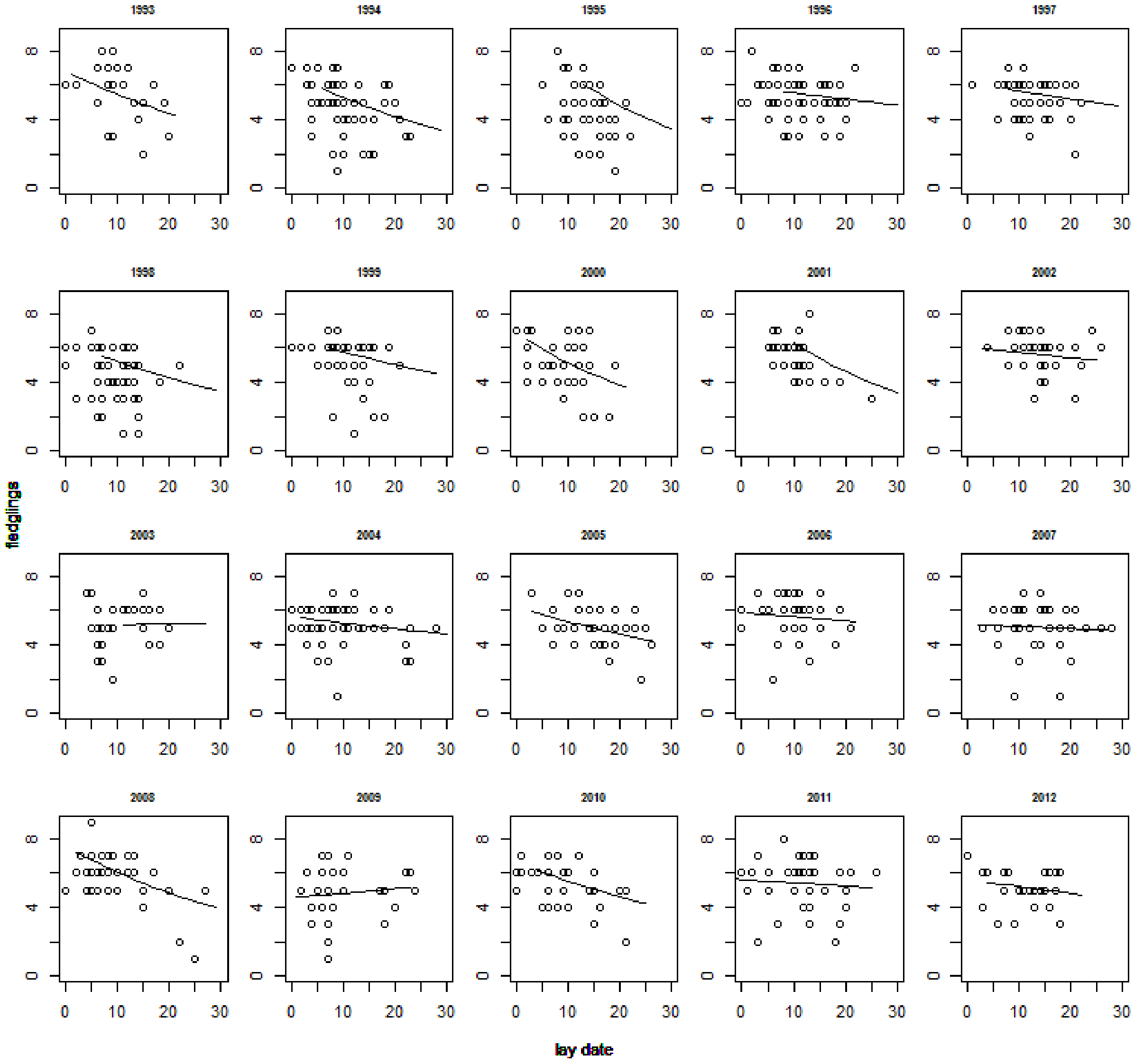

Fig. S5. Seasonal patterns of number of fledglings from successful nests across 20 years. Lines show predicted number of fledglings from a GLM with Poisson distribution (log link) without covariates. Lay dates are shown as relative to the earliest lay date in each year.

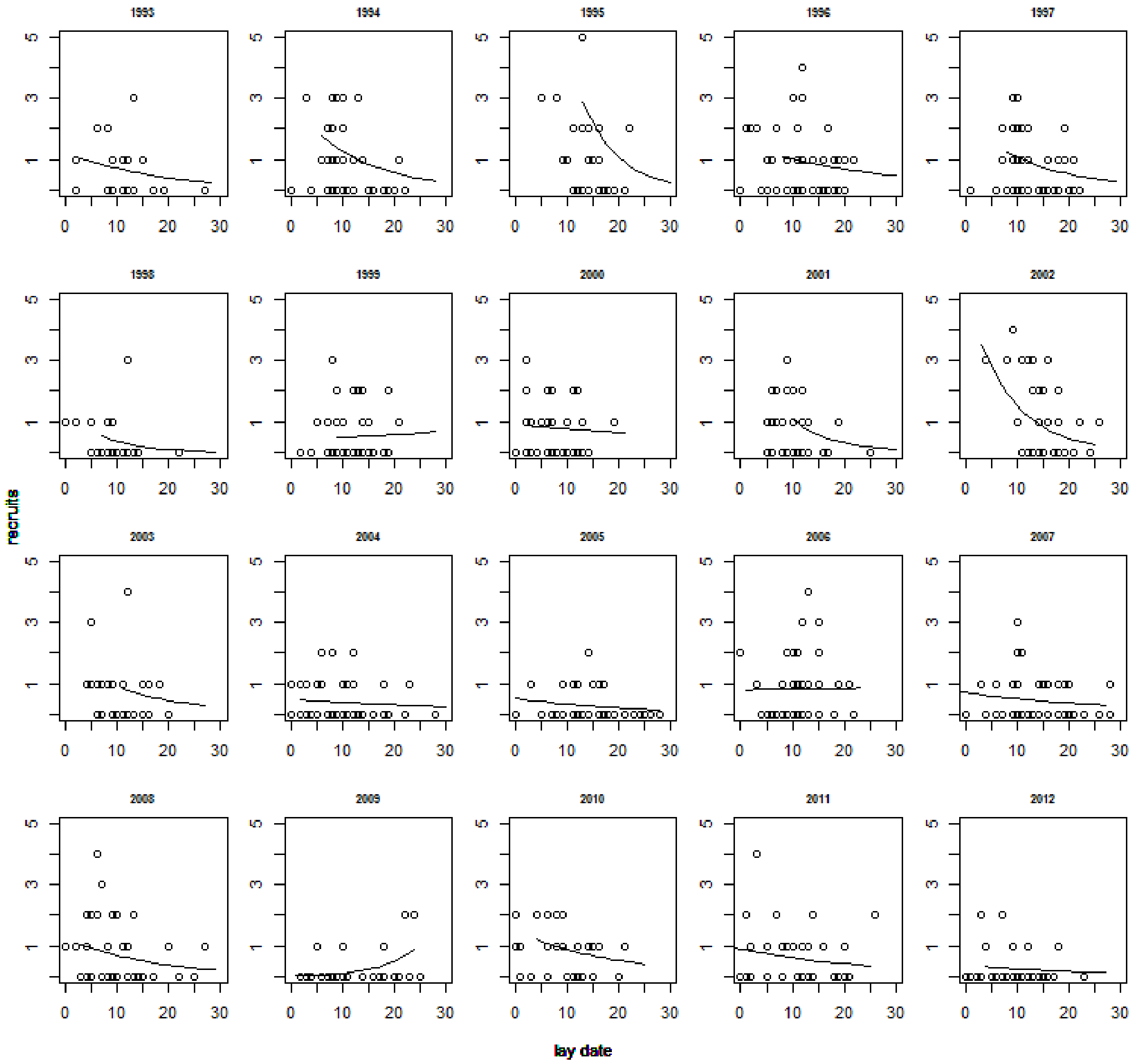

Fig. S6. Seasonal patterns of number of recruits (including failed nests) across 20 years. Lines show predicted number of recruits from a GLM with Poisson distribution (log link) without covariates. Lay dates are shown as relative to the earliest lay date in each year.

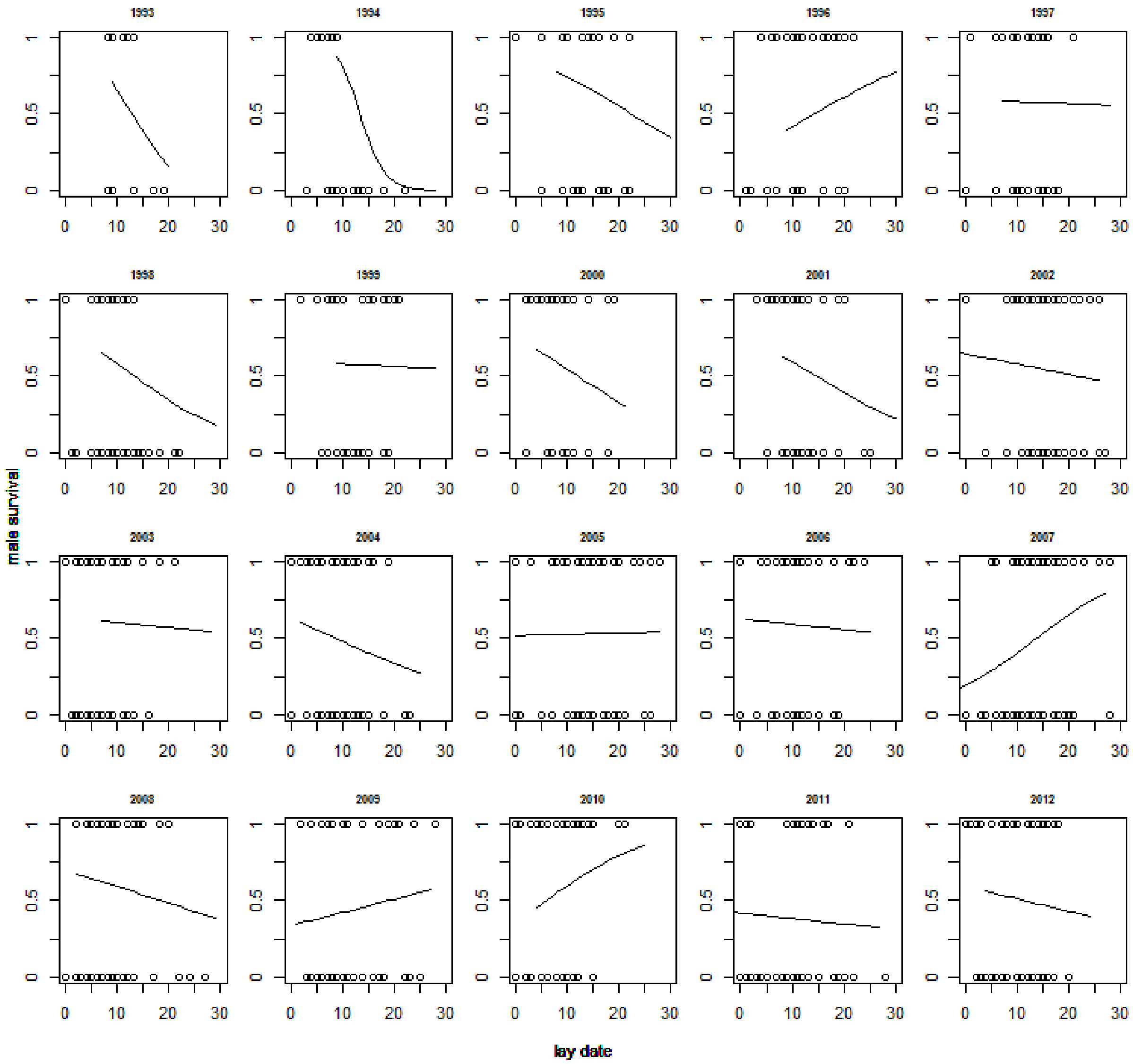

Fig. S7. Seasonal patterns of male survival across 20 years. Lines show predicted male survival from a GLM with binomial distribution (logit link) without covariates. Lay dates are shown as relative to the earliest lay date in each year.

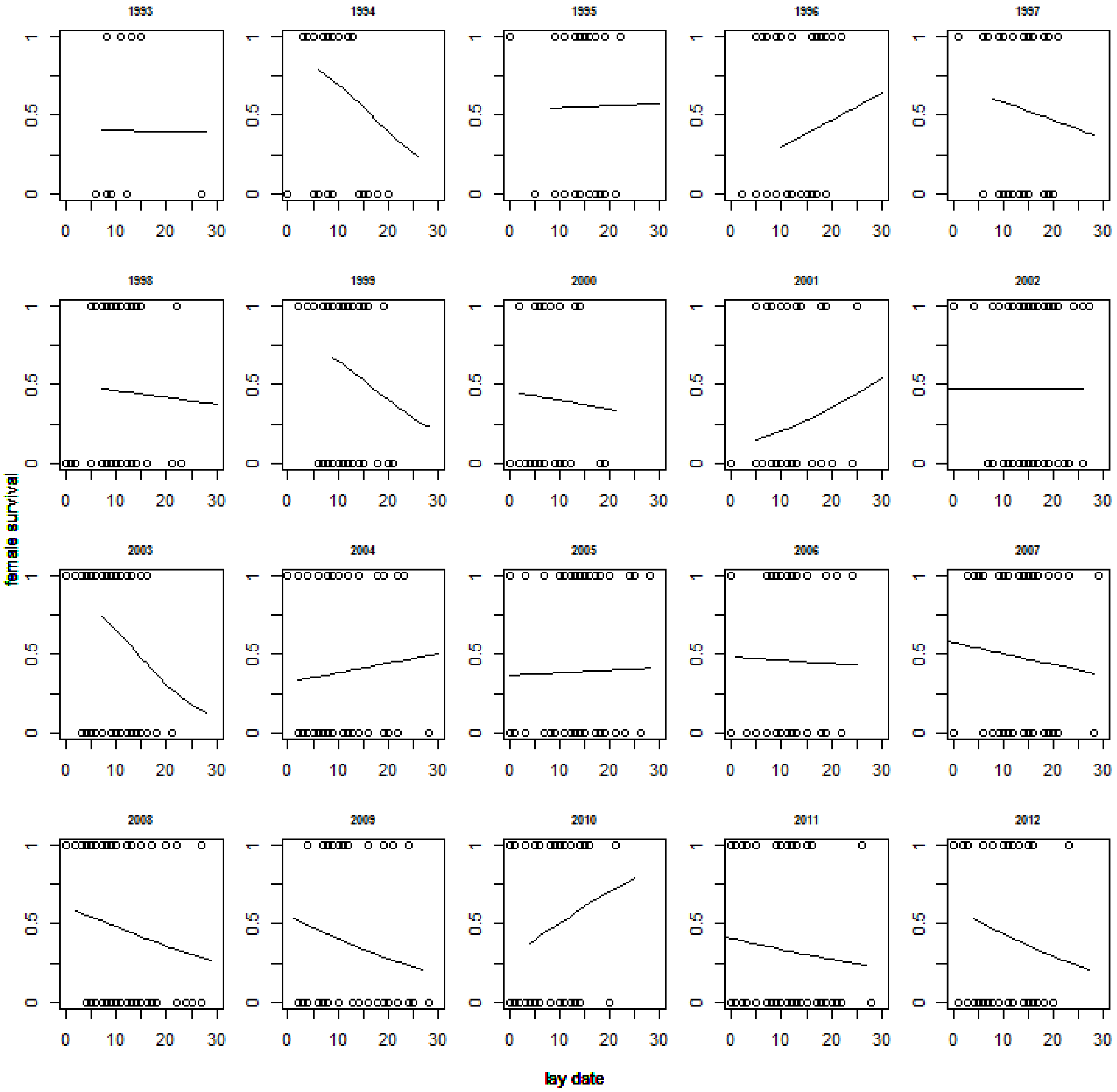

Fig. S8. Seasonal patterns of female survival across 20 years. Lines show predicted female survival from a GLM with binomial distribution (logit link) without covariates. Lay dates are shown as relative to the earliest lay date in each year.

4.2 Extended results for temporal trends of within-season fitness pattern

Temporal trends of selection for breeding time - analyses without covariates
Selection for breeding time as measured by the seasonal decline in reproductive and survival parameters, i.e. the relationship between fitness and breeding date, varied between years (Fig. S9). For all demographic rates except survival of adult females the slopes of the relationship between fitness and breeding date tended to have changed from distinct negative slopes in the early years to less negative or even positive slopes in more recent years. Results from the data subset only containing successful nests were qualitatively similar (Table S1).

Table S1. Estimated temporal trends for within-season fitness patterns of wheatears (weighted linear regression: slope~year, $w=1/SE_{slope}$, N=20 years, df=19). Within-season slopes of the relationship between demographic rates and breeding time were estimated using data from successful nests only (first nest attempts), without covariates. See Supporting Information 1 'Extended Methods' for analysis details.

	**estimate±SE**	**t**	**p**	**R^2**
fledglings	0.0009±0.0004	2.28	0.035	0.224
recruits	0.0026±0.0021	1.22	0.239	0.076
male survival	0.0052±0.0032	1.59	0.129	0.123
female survival	-0.0016±0.0026	-0.630	0.54	0.022

Temporal trends of selection for breeding time - analyses with covariates
The slopes of the relationships between fitness and breeding time can also change due to other factors than food abundance (e.g. rain, Tarwater & Beissinger 2013, Brown et al. 2013). At our study location rainfall often showed seasonal trends (i.e. with more days with rainfall during the nestling period either for late breeding, or for early breeding wheatears). There was, however, no evidence for a temporal trend in annual slopes from linear regression of rainfall against lay date (rainfall analysed as number of days with rainfall >0 mm during the nestling period; weighted regression: year estimate=0.0031±0.0091 SE, t=0.34, p=0.74).

When accounting for covariates (female age, territory field layer height, number of rain days during nestling period) a temporal trend for slopes of the within-season relationship between fitness and breeding time was apparent only for recruits when using data from failed and successful attempts (Fig. S10). Using data from successful nests only we found no year trend for any of the demographic rates (all p>0.4).

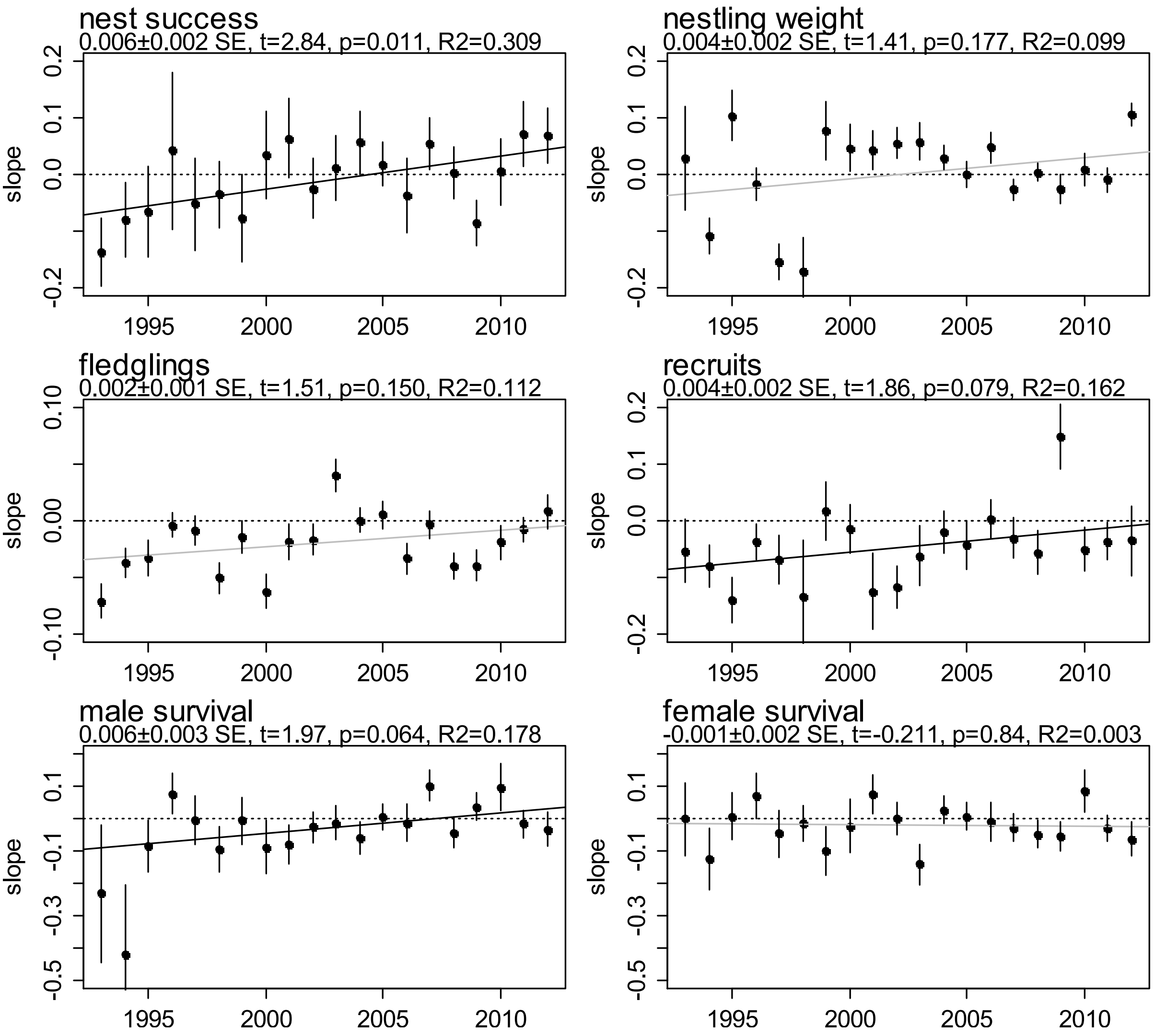

Fig. S9. Slope estimates and their SE for the within-season relationships between fitness and breeding date across the 20 year study period using data from all (i.e. including failed) breeding attempts; estimated **without covariates**. Year trends were analysed by weighted regression: slope ~ year, $w=1/SE_{slope}$, N=20 years), and year estimate with associated statistics are presented on top of each panel. Year trends with p-values ≤0.1 are shown by black lines. Dotted line marks zero slope. See Methods (main text) for details.

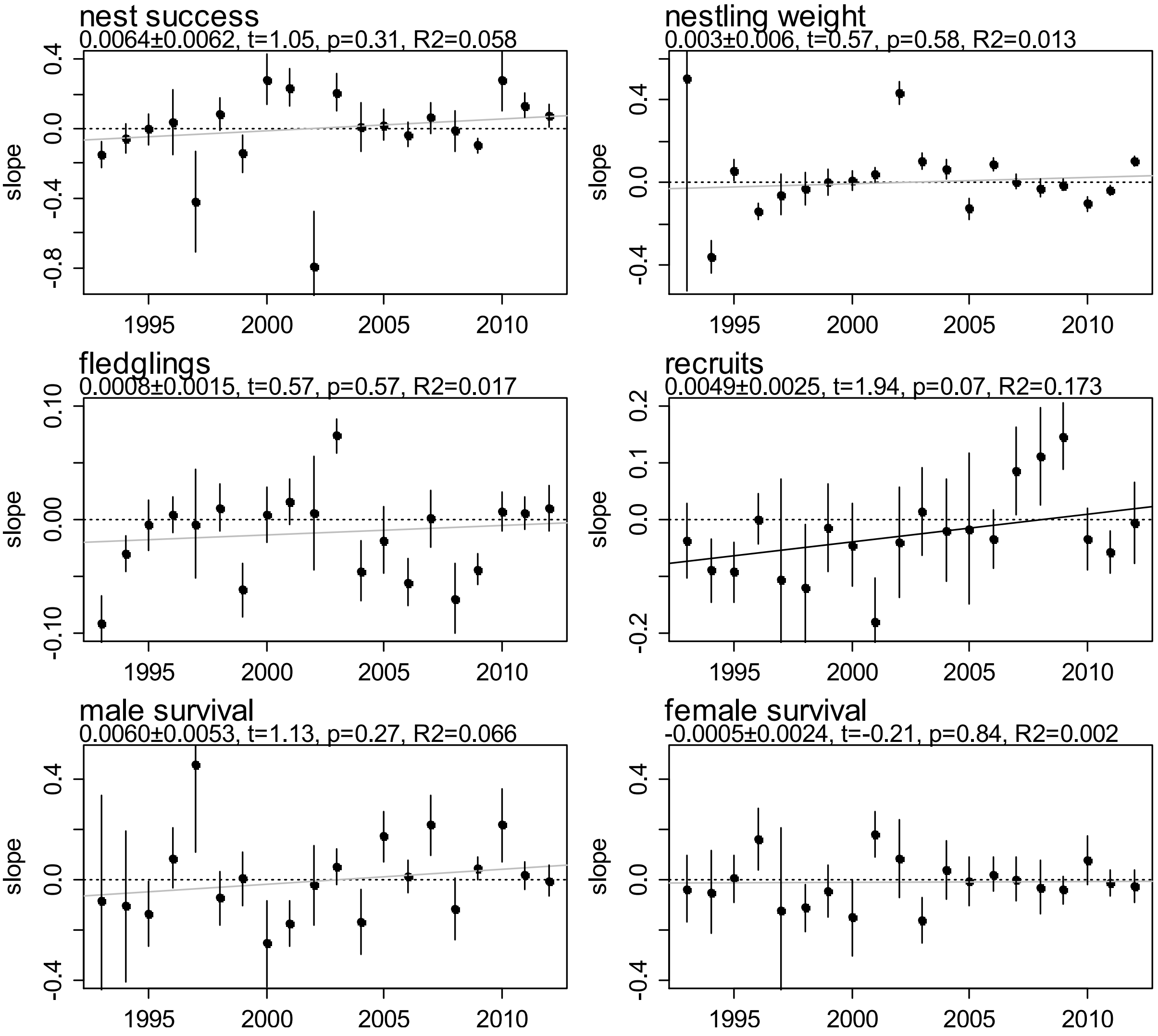

Fig. S10. Slope estimates and their SE for the within-season relationships between fitness and breeding date across the 20 year study period using data from all (i.e. including failed) breeding attempts; estimated **accounting for covariates** age (first year or older) of the breeding female, territory field layer height (short or tall), and number of days with rainfall during the nestling period. Year trends were analysed by weighted regression: slope ~ year, $w=1/SE_{slope}$, N=20 years), and year estimate with associated statistics are presented on top of each panel. Year trends with p-values ≤0.1 are shown by black lines. Dotted line marks zero slope. See Methods (main text) for details.

References

Tarwater CE & Beissinger SR 2013. Opposing selection and environmental variation modify optimal timing of breeding. P Natl Acad Sci USA 110: 15365–15370.

Brown CR et al. 2013. Fluctuating viability selection on morphology of cliff swallows is driven by climate. J Evol Biol 26: 1129–1142.
